# Hypoxia-Associated Glycolytic–Lipogenic Reprogramming and Impaired Antigen Presentation in SPP1^+^ Tumor-Associated Macrophages in Ovarian Cancer

**DOI:** 10.3390/ijms27219904

**Published:** 2026-11-05

**Authors:** Xiaohu Zuo, Shouqi Huang, Yingge Li, Zhi Wang, Hongyang Xue, Jie Min, Li Hong, Suting Li

**Affiliations:** 1 Department of Gynecology and Obstetrics, Renmin Hospital of Wuhan University, Wuhan 430060, China; m13277038971_2@163.com (X.Z.); 2020305231018@whu.edu.cn (S.H.); wangzhi6666@whu.edu.cn (Z.W.); 2018305230034@whu.edu.cn (H.X.); jie_min@whu.edu.cn (J.M.); 2 Cancer Center, Renmin Hospital of Wuhan University, Wuhan 430060, China; liyingge@whu.edu.cn

**Keywords:** ovarian cancer, SPP1^+^ tumor-associated macrophages, metabolic reprogramming, hypoxia, HIF1A, single-cell transcriptomics, spatial transcriptomics

## Abstract

Ovarian cancer has an immunosuppressive microenvironment in which SPP1^+^ tumor-associated macrophages (TAMs) are linked to poor prognosis and immune evasion, yet their molecular features and metabolic mechanisms remain unclear. We therefore set out to determine whether SPP1 marks a discrete TAM subpopulation or a functional state in treatment-naïve high-grade serous ovarian cancer (HGSOC), to define the transcriptional and metabolic program that accompanies it, and to test whether local hypoxia is associated with it. Tumor-derived, treatment-naïve samples from three single-cell datasets (one lesion per patient; 47 patients) yielded 385,504 cells; Seurat v5 and Harmony were used for clustering, annotation, and batch correction. SPP1^+^/SPP1^−^ macrophages were compared by differential expression, GO/KEGG/GSEA, metabolic scoring, and transcription factor inference, and the definition was retested with within-sample SPP1-high versus SPP1-low comparisons and with MSigDB Hallmark gene sets scored by two independent algorithms. Validation used Visium spatial transcriptomics (GSE263920), TCGA-OV (n = 429), CD8^+^ T cell exhaustion analysis, CellChat and a syngeneic ID8 model. Of 64,067 macrophages (16.62%), 82.0% were SPP1^+^; upregulated genes were enriched in glycolysis, monocyte migration and tissue remodeling, whereas MHC class II and neutrophil-related genes were downregulated. At the sample level, glycolysis, fatty acid synthesis and glutaminolysis scores were elevated and the cholesterol synthesis score was reduced, with no evidence of suppressed fatty acid oxidation or oxidative phosphorylation; HIF1A ranked 4th and NR1H3 3rd among 271 transcription factors. All 8 spatial sections showed positive hypoxia–SPP1 correlations, and hotspots showed elevated glycolysis, EMT, immunosuppression and macrophage scores. No robust association was found between SPP1^+^ TAM infiltration and CD8^+^ T cell exhaustion (*p* = 0.315). MDK–LRP1/SDC1 was the major macrophage-directed input. Immunofluorescence showed SPP1 immunoreactivity in a subset of CD68^+^ macrophages within HIF1α-rich areas of ID8 tumors, without CD8^+^PD-1^+^ T cell aggregation. SPP1^+^ TAMs therefore correspond to a hypoxic functional state with HIF1A activation, a glycolytic–lipogenic transcriptional program, reduced cholesterol synthesis gene expression and a reduced MHC class II program; protein-level and functional validation, and direct tests of HIF1A dependency, remain necessary.

## 1. Introduction

Ovarian cancer is the most lethal gynecological malignancy, with global epidemiological data indicating more than 300,000 new cases and approximately 200,000 deaths annually [1]. Although cytoreductive surgery combined with platinum-based chemotherapy remains the current standard of care, the long-term prognosis of patients with advanced disease is still unsatisfactory, with a 5-year survival rate below 40%, and chemoresistance and tumor recurrence represent critical unresolved clinical challenges [2,3]. Immune checkpoint blockade therapy has achieved considerable efficacy in multiple solid tumors; however, the response rate of ovarian cancer patients to such therapy is only 10–15% [4]. This phenomenon is closely associated with the “cold tumor” immune phenotype of ovarian cancer, which is characterized by insufficient numbers of tumor-infiltrating lymphocytes, cytotoxic T cell exhaustion, and massive accumulation of immunosuppressive cells within tumor lesions.

Tumor-associated macrophages (TAMs) are among the most abundant immune cell populations in the ovarian cancer microenvironment, accounting for more than 50% of infiltrating immune cells [5,6,7]. TAMs exhibit high heterogeneity, and recent single-cell studies have revealed increasingly refined subpopulation classifications [8,9]. Among these, SPP1^+^ TAMs have been identified as a distinct pro-tumorigenic subpopulation closely associated with poor prognosis, chemoresistance, and immunosuppression across multiple solid tumors [10,11,12]. Hou et al. integrated six high-grade serous ovarian cancer single-cell datasets and identified that the SPP1^+^ TAM subpopulation drives T cell exhaustion through the SPP1–CD44 axis, and that blockade of this axis restores CD8^+^ T cell function and inhibits tumor growth [13]. Luo et al. identified PLIN2^hi macrophages enriched in ovarian cancer ascites that promote tumor metastasis via the HIF1α/SPP1 signaling axis, directly linking lipid metabolism to the hypoxic microenvironment [14]. Together, these studies establish the central position of SPP1^+^ TAMs in the immunosuppressive network of ovarian cancer.

SPP1 (secreted phosphoprotein 1, also known as osteopontin) is a multifunctional cytokine involved in cell adhesion, migration, and immune regulation [15]. In the tumor microenvironment, SPP1^+^ TAMs drive tumor progression through mechanisms including SPP1 secretion, promotion of angiogenesis, induction of T cell exhaustion, and recruitment of myeloid-derived suppressor cells [10,11]. Recent studies have further revealed that the differentiation and maintenance of SPP1^+^ macrophages are strongly influenced by intercellular interactions and hypoxic conditions within the tumor microenvironment, and that these cells can form a physical barrier through interaction with cancer-associated fibroblasts, restricting CD8^+^ T cell infiltration into the tumor core [11]. However, the detailed molecular features, metabolic reprogramming mechanisms, and spatial distribution patterns of SPP1^+^ TAMs in ovarian cancer remain incompletely understood, and existing studies are largely limited to a single dataset or a single analytical level, lacking systematic validation across datasets and technical platforms.

The objective of this study was therefore threefold: first, to determine whether SPP1 expression in high-grade serous ovarian cancer (HGSOC) macrophages marks a discrete subpopulation or a continuous functional state; second, to define the transcriptional, metabolic and antigen-presentation program that accompanies this state in treatment-naïve tumors; and third, to test whether local hypoxia and HIF1A activity are spatially and quantitatively associated with it across single-cell, tissue-spatial and patient-level scales. To address these questions we integrated treatment-naïve single-cell transcriptomes from tumor tissue only, one lesion per patient, spatial transcriptomics of untreated HGSOC, the TCGA-OV cohort, cell–cell communication inference and a syngeneic ID8 tumor model. We deliberately framed the work as a hypothesis-generating, multi-level observational study: the analyzes define the molecular program of SPP1^+^ TAMs and the strength of its association with hypoxia, and they specify which parts of the model still require direct functional and protein-level testing.

## 2. Results

### 2.1. Construction of a Single-Cell Atlas of Treatment-Naïve Ovarian Cancer Samples

**Figure 1:**
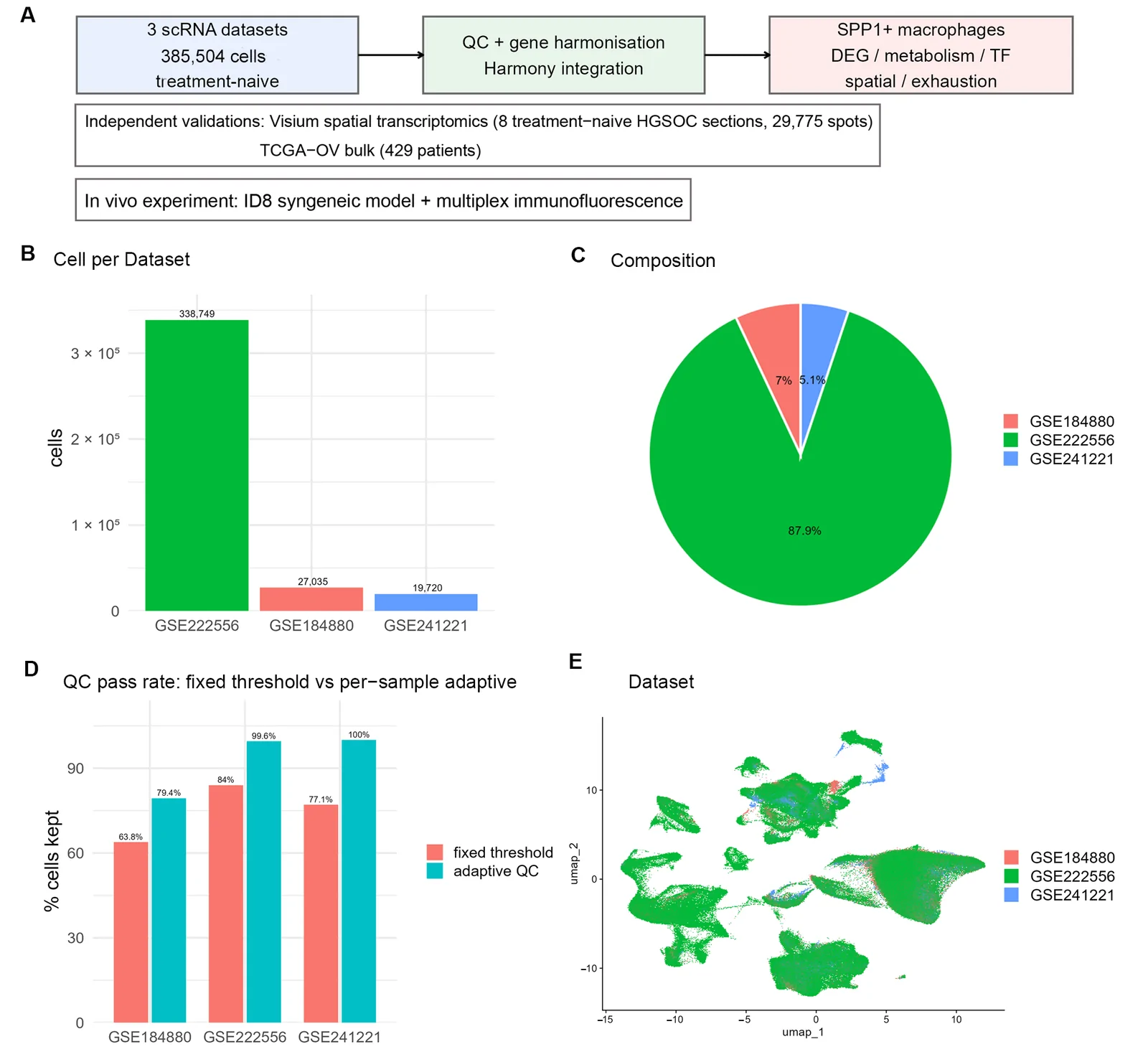
Study design and quality control. (**A**) Study design workflow; (**B**) cells analyzed per dataset; (**C**) composition of the atlas by dataset; (**D**) cell numbers and retention rates under per-sample adaptive quality control versus conventional fixed thresholds; (**E**) UMAP colored by dataset.

To dissect the cellular heterogeneity of the ovarian cancer microenvironment while excluding both treatment effects and non-malignant tissue, we integrated treatment-naïve samples from three public single-cell datasets (GSE184880, GSE222556, GSE241221). Only tumor-derived samples were retained: the five normal ovarian samples of GSE184880 were removed, and where a patient contributed more than one lesion in GSE222556 a single lesion was kept, so that every patient contributed exactly once (Table 1, Supplementary Table S1). The final atlas comprised 398,665 cells from 47 samples of 47 patients; after per-sample adaptive quality control, 385,504 cells entered downstream analysis (Table 1, Figure 1A–C).

For comparison, when the complete deposited series of each dataset were analyzed, before the exclusion of non-malignant samples and duplicate lesions, conventional fixed thresholds retained only 392,500 cells (81.0%), with retention rates of 84.0% for GSE222556 and only 63.8% for GSE184880; the unified adaptive criteria raised these to 99.5% and 79.4%, respectively (Figure 1C, Supplementary Table S1). In the tumor-only set analyzed here the adaptive criterion retained 69.7% of GSE184880 cells and 99.6% of GSE222556 cells (Table 1). Fixed thresholds therefore systematically disadvantage datasets with high annotation depth.

**Figure 2:**
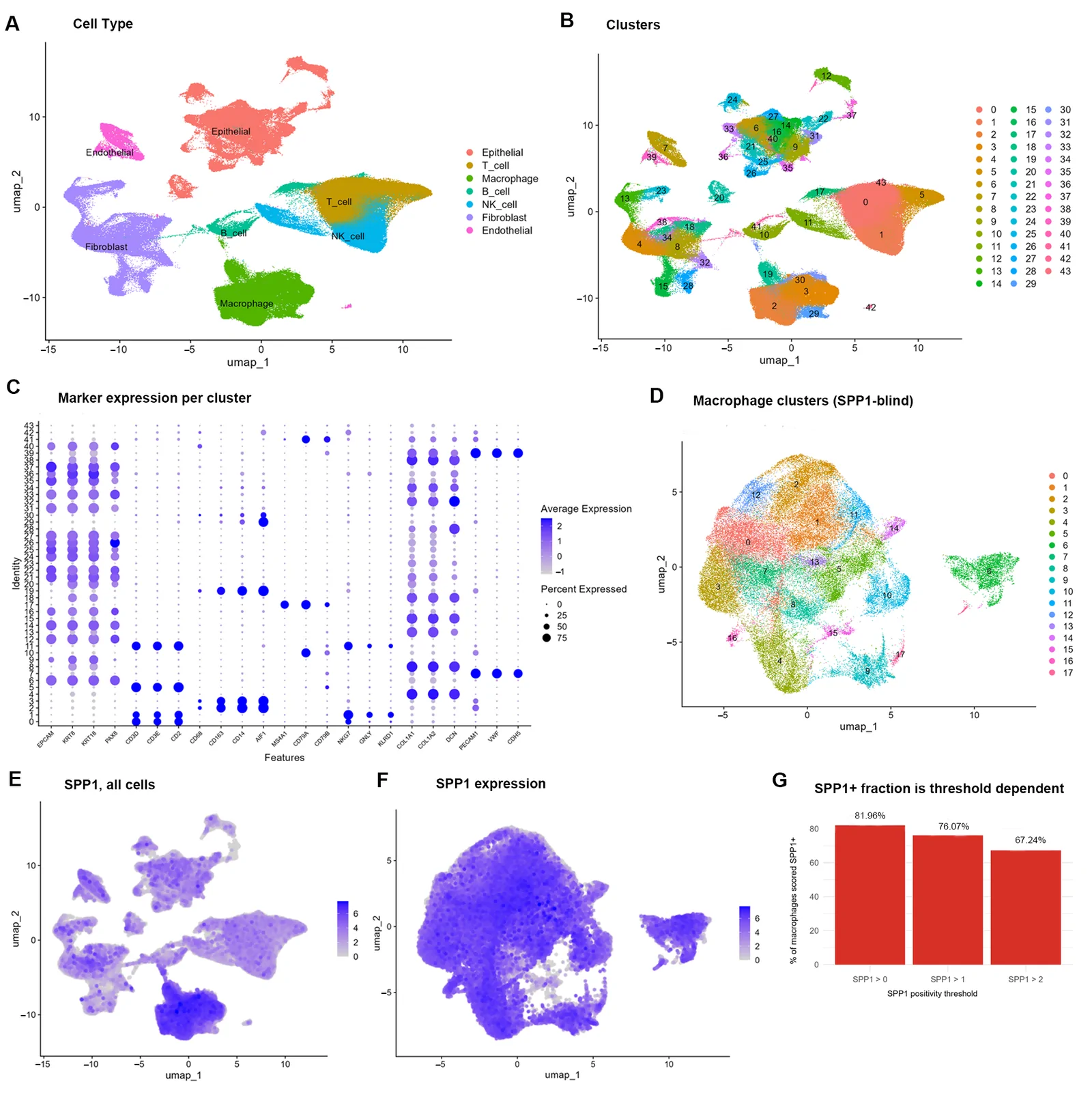
Single-cell atlas of ovarian cancer and definition of SPP1^+^ TAMs. (**A**) UMAP of the seven cell types; (**B**) UMAP of the Louvain clusters; (**C**) dot plot of classical marker genes for each cell type; (**D**) UMAP of 18 macrophage subpopulations; (**E**) SPP1 expression across all cells; (**F**) SPP1 expression in macrophages; (**G**) variation in the SPP1^+^ proportion across thresholds.

After Harmony correction, Uniform Manifold Approximation and Projection (UMAP) showed that the three datasets were well mixed (Figure 1D). Based on classical marker genes, seven cell types were annotated (Figure 2A–C, Table 2): T cells, 94,463 (24.50%); epithelial cells, 87,474 (22.69%); fibroblasts, 65,039 (16.87%); macrophages, 64,067 (16.62%); NK cells, 44,638 (11.58%); B cells, 17,193 (4.46%); and endothelial cells, 12,630 (3.28%). The marker gene specificity of each cell type was clear (Figure 2C). A small number of rare clusters were still predominantly derived from a single dataset, indicating residual batch structure under Louvain clustering; these clusters were not given independent biological interpretation.

### 2.2. Macrophage Subpopulation Structure and the SPP1 Expression Landscape

All 64,067 macrophages (16.62% of all cells) were extracted and re-clustered, yielding 18 macrophage subpopulations (Figure 2D). SPP1 showed pronounced focal expression on the UMAP (Figure 2E,F). Using SPP1 > 0 as the reference criterion, 52,512 SPP1-expressing macrophages were identified, an expression positivity rate of 81.96% (Figure 2G, Table 3). This is a detection rate rather than a subpopulation frequency: the positivity rates were 6.34% (GSE184880), 87.04% (GSE222556) and 6.63% (GSE241221) and varied in the same direction as the median sequencing depth of each dataset (Supplementary Table S2).

**Figure A1:**
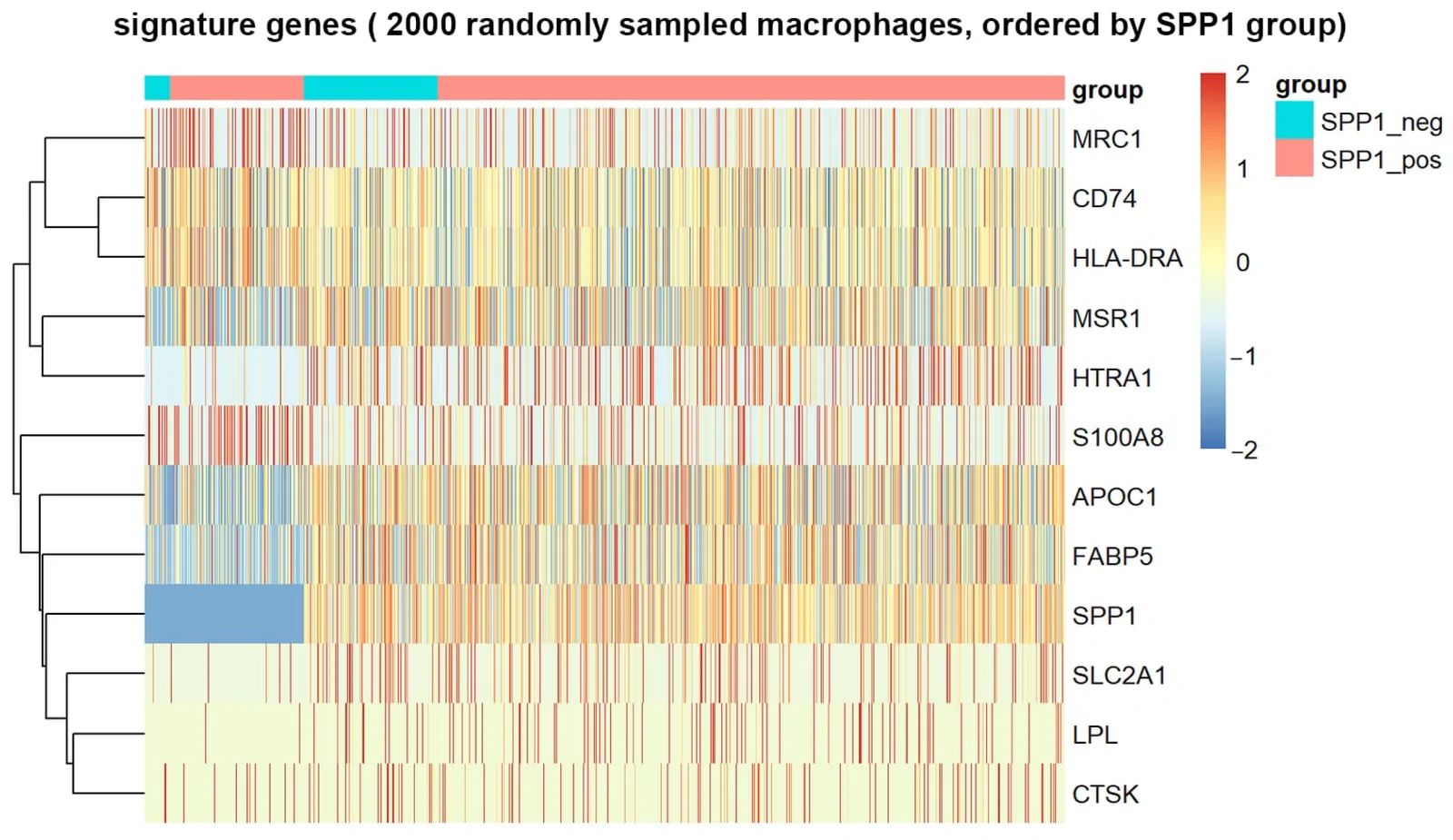
Heatmap of SPP1^+^ TAM signature genes.

Importantly, SPP1^+^ cells were not confined to a single subpopulation: the proportion of SPP1^+^ cells across subpopulations ranged from 28.98% (subpopulation 15) to 100.0% (subpopulation 16) (Supplementary Table S3). SPP1 is therefore better regarded as a marker of a functional state than as a marker of a discrete subpopulation, and the groups compared here were defined by SPP1 expression rather than by cluster assignment. The grouping corresponded to a consistent separation of expression at the single-cell level (Figure A1).

Threshold sensitivity analysis showed that raising the cut-off to SPP1 > 1 and SPP1 > 2 gave positivity rates of 76.07% and 67.24%, respectively (Table 3). Even with the most stringent threshold, two thirds of macrophages remained positive, indicating a high background of SPP1 detection in this compartment; the focus of this study is therefore not the absolute proportion of the population but the consistency of the molecular features and downstream associations across definitions. To test that consistency directly, the principal comparisons were repeated with a definition that is internal to each sample.

### 2.3. Robustness of the SPP1 Definition

Because SPP1 positivity is threshold dependent, the principal comparisons were repeated with a definition that is internal to each sample. Within each sample, macrophages were ranked by SPP1 expression and the top and bottom thirds were compared, so that both groups come from the same tumor and the same sequencing library. Under this definition 695 genes were differentially expressed (449 up, 246 down), and 69.7% of the genes upregulated under the reference definition and 59.3% of those downregulated were recovered (Jaccard index 0.528; Supplementary Table S4). The direction of the metabolic differences was preserved (Supplementary Table S5): glycolysis was higher in 44 of 44 samples (*p* = 1.14 × 10^−13^), cholesterol synthesis was lower in 38 of 44 (*p* = 1.32 × 10^−7^), and the fatty acid synthesis score remained higher (*p* = 1.14 × 10^−13^). The glutaminolysis difference, which was modest under the reference definition, did not reach significance under the within-sample definition (*p* = 0.212). The same pattern was obtained when the split was made at the sample median. The molecular features described here therefore do not depend on the particular SPP1 cut-off, although the absolute positivity rate does.

### 2.4. Molecular and Functional Features of SPP1^+^ TAMs

**Figure 3:**
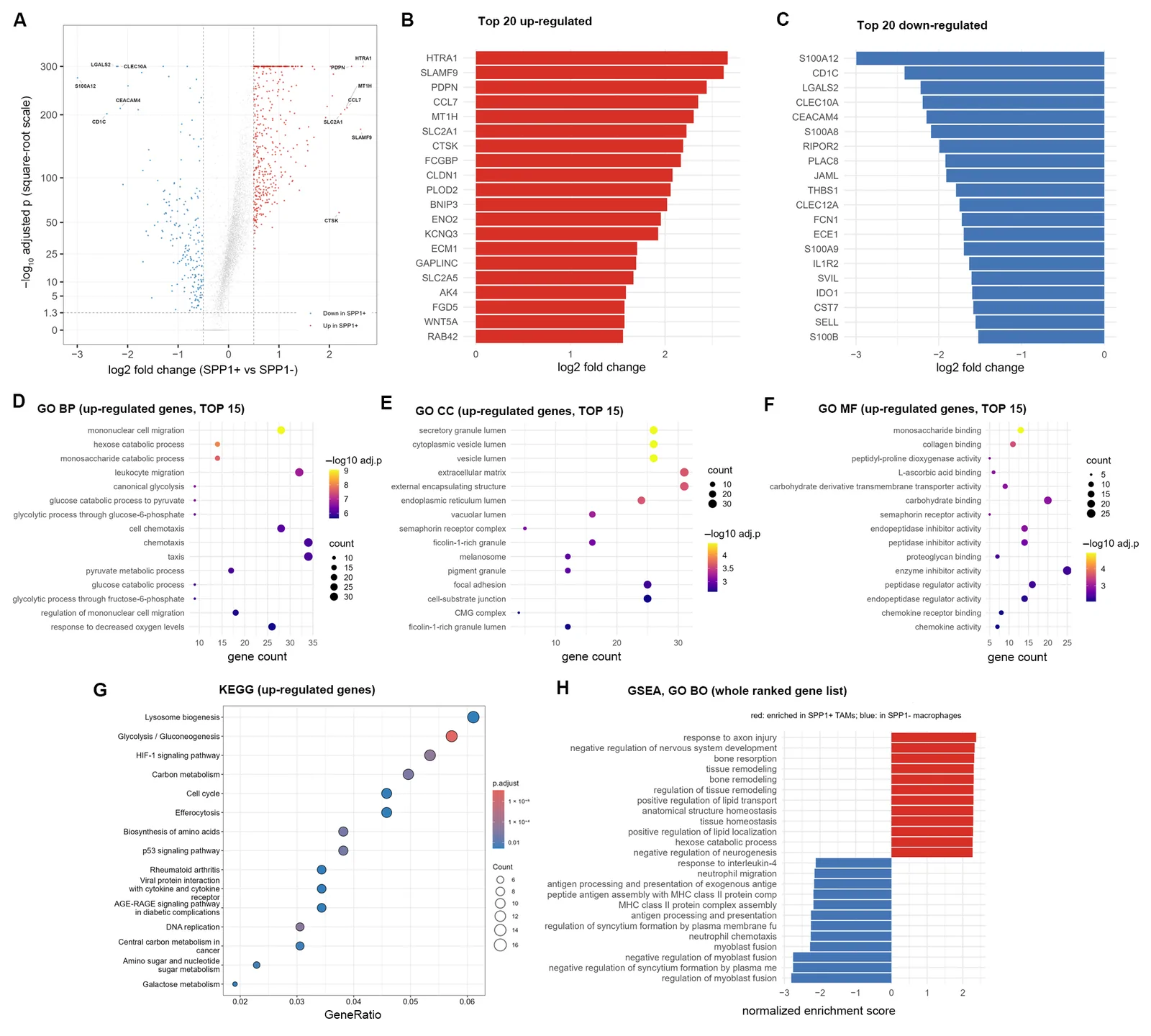
Differential expression and pathway enrichment in SPP1+ TAMs. (**A**) Volcano plot; (**B**) top 20 upregulated genes; (**C**) top 20 downregulated genes; (**D**–**F**) GO enrichment (biological process, cellular component, molecular function); (**G**) KEGG pathway enrichment; (**H**) GSEA based on the ranking of all tested genes.

Differential expression analysis between SPP1^+^ and SPP1^−^ macrophages identified 425 upregulated and 208 downregulated genes (Figure 3A–C; Supplementary Table S4). Among the upregulated genes (Figure 3B), in addition to genes directly related to the hypoxia response (SLC2A1, ENO2, BNIP3, PLOD2, SLC2A5), these included matrix-remodeling genes (HTRA1, PDPN, CTSK, CLDN1, ECM1, WNT5A, FCGBP), the metallothionein MT1H, the chemokine CCL7, lipid transport and metabolism genes (LPL, SCD, APOC1, FABP5) and the scavenger receptor MSR1. Glycolysis genes HK2, PKM and LDHA were synchronously upregulated, as were the proliferation genes MKI67 and TOP2A. Immunosuppressive molecules MARCO and IL10 were only mildly upregulated. Among the downregulated genes (Figure 3C), neutrophil-related genes S100A12, S100A8, S100A9 and SELL were significantly reduced, and the MHC class II genes HLA-DRA, HLA-DRB1 and CD74 together with their transcriptional regulator CIITA were all significantly downregulated. This pattern indicates that the MHC class II transcriptional program is reduced in SPP1^+^ TAMs; it does not by itself demonstrate impaired antigen presentation, because surface protein levels, peptide loading and the capacity to stimulate T cells were not measured. Notably, the M2-associated marker MRC1 was not upregulated in SPP1^+^ TAMs and IDO1 was significantly downregulated, so these cells cannot be assigned to the M2 category on the basis of these markers.

Gene Ontology (GO) enrichment analysis showed that upregulated genes were significantly enriched in biological processes related to glucose metabolism, myeloid cell migration, and hypoxia response (Figure 3D–F, Supplementary Table S6). Kyoto Encyclopedia of Genes and Genomes (KEGG) pathway enrichment analysis (Figure 3G, Supplementary Table S7) revealed that the most significant pathway was glycolysis/gluconeogenesis, followed by carbon metabolism, amino acid biosynthesis, DNA replication, and HIF-1 signaling pathway; cholesterol metabolism and lysosome biogenesis were also enriched. The HIF-1 pathway corroborates the hypoxia axis examined below, and the direction of the cholesterol metabolism result is consistent with the metabolic phenotype. Gene set enrichment analysis (GSEA) utilized the ranking information of all tested genes (Figure 3H, Supplementary Table S8). Gene sets enriched in SPP1^+^ TAMs included tissue remodeling, wound response, metal ion stress response, and regulation of anatomical structure morphogenesis, indicating that matrix remodeling is a coordinately upregulated module. In contrast, gene sets enriched in SPP1^−^ macrophages centered on antigen processing and presentation, consistent with the significant downregulation of S100A8/A9/A12 and MHC-II genes.

### 2.5. Metabolic Reprogramming Features of SPP1^+^ TAMs

**Figure 4:**
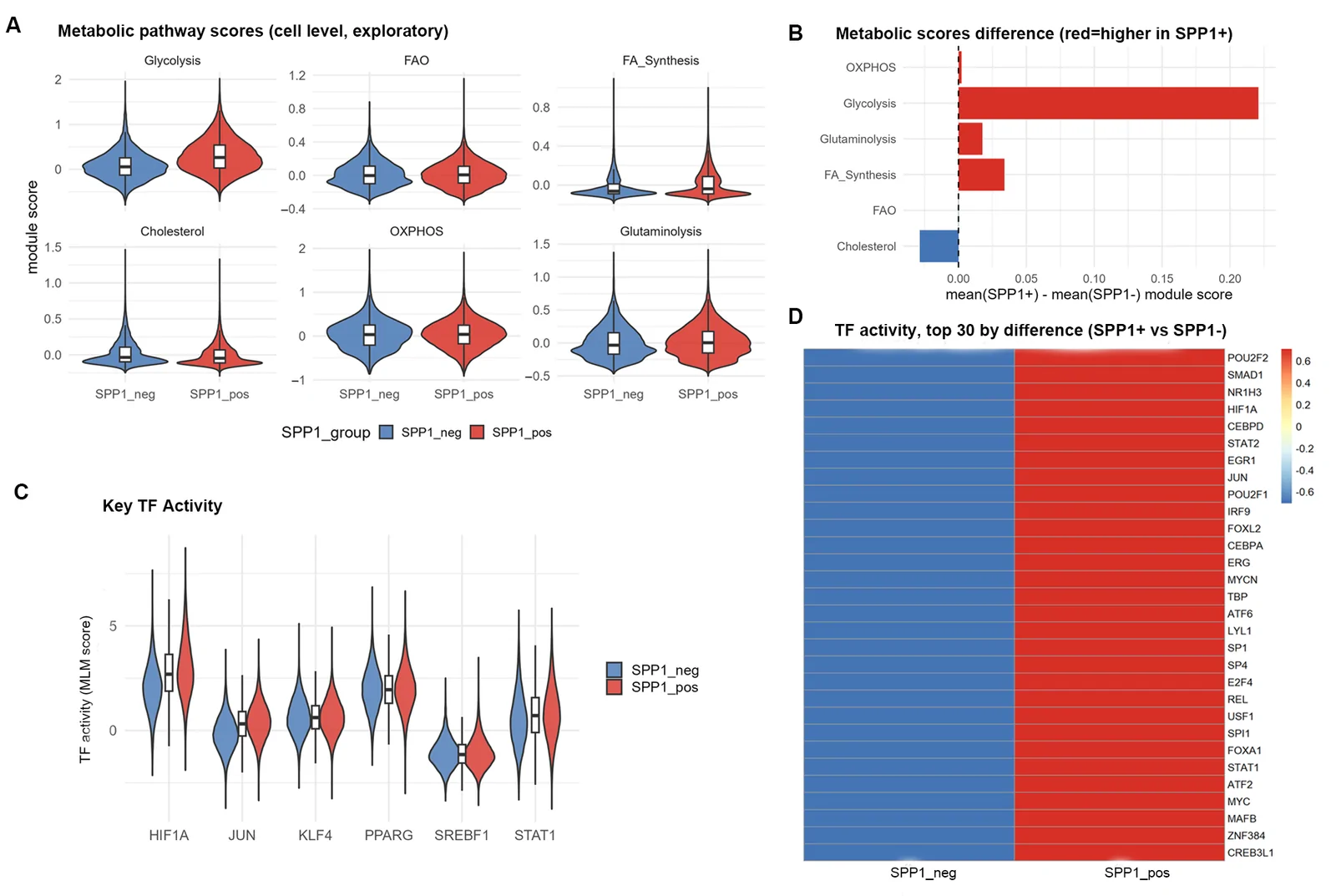
Metabolic reprogramming and transcriptional regulation of SPP1^+^ TAMs. (**A**) Distribution of module scores for the six metabolic pathways; (**B**) cell-level and sample-level between-group differences in metabolic scores; (**C**) activities of key transcription factors; (**D**) heatmap of the 30 transcription factors with the greatest activity changes.

Based on the differential expression and enrichment results, the activities of six core metabolic pathways were evaluated (Figure 4A,B, Table 4). Because cell-level testing suffers from pseudoreplication, sample-level results (47 paired samples) were used for inference. The glycolysis score of SPP1^+^ macrophages was higher than that of SPP1^−^ macrophages in 47 of 47 samples (median difference 0.233, *p* = 1.42 × 10^−14^), consistent with the coordinated upregulation of SLC2A1, HK2, PKM and LDHA. The fatty acid synthesis score was higher in 47 of 47 samples (median difference 0.036, *p* = 1.42 × 10^−14^), with SCD and the fatty acid transport genes LPL, APOC1 and FABP5 upregulated, indicating that lipid uptake and de novo synthesis are simultaneously active. The glutaminolysis score was higher in 31 of 47 samples (median difference 0.013, *p* = 0.0216), suggesting that glutamine replenishes the TCA cycle. In contrast, the cholesterol synthesis score was lower in SPP1^+^ than in SPP1^−^ macrophages in 41 of the 47 samples (median difference −0.021, *p* = 3.52 × 10^−7^); the direction was reversed in the remaining 6 samples (Table 4), and LDLR and SQLE were downregulated. No suppression of fatty acid oxidation or oxidative phosphorylation was observed: the fatty acid oxidation score was higher in 29 of 47 samples but did not reach significance (*p* = 0.057), and the oxidative phosphorylation score did not differ significantly at the sample level (*p* = 0.363). These scores are transcriptional signatures of small enzymatic modules, not measurements of metabolic flux; the direction of the glycolysis, fatty acid metabolism and oxidative phosphorylation differences was recovered with MSigDB Hallmark gene sets and with AUCell scoring, whereas the cholesterol result depends on whether efflux genes are included in the set (Supplementary Table S9).

In summary, at the transcriptional level SPP1^+^ TAMs show increased glycolysis, increased lipid uptake and synthesis gene expression and reduced cholesterol synthesis gene expression. This combination does not match the classical M1/M2 dichotomy, but it should be described as a transcriptional metabolic signature rather than as a metabolic reprogramming until protein and flux measurements are available.

### 2.6. Curated Gene Sets and Independent Scoring Confirm the Transcriptional Phenotype

**Figure A2:**
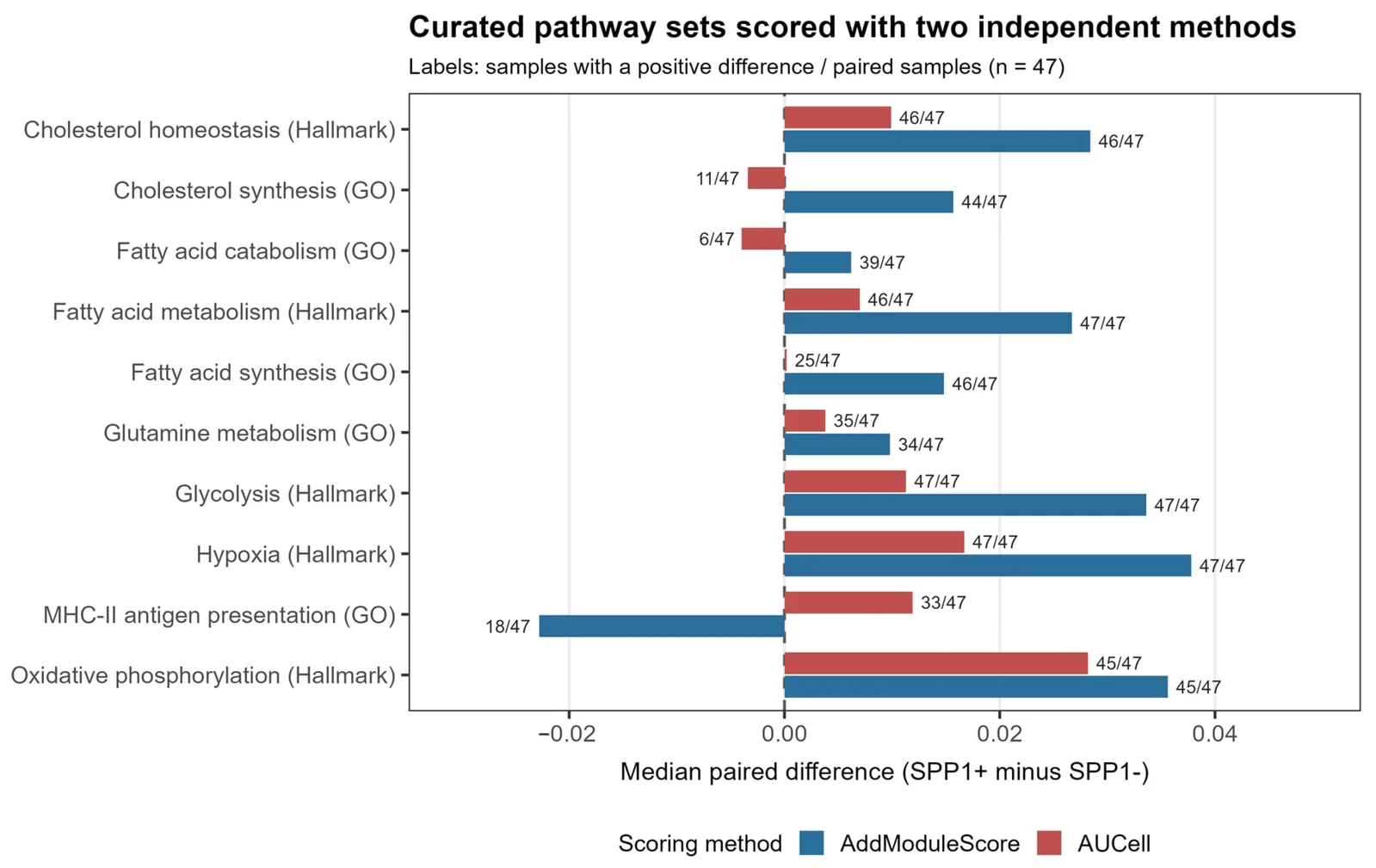
Pathway scores obtained with established curated gene sets and two independent scoring algorithms. Each curated set (MSigDB Hallmark or GO biological process) was scored with AddModuleScore and with AUCell; bars show the median paired difference between SPP1^+^ and SPP1^−^ macrophages across samples, and labels give the number of samples with a positive difference over the number of paired samples.

To test whether these findings depend on the small, author-defined gene sets, all pathways were re-scored with established curated sets (MSigDB Hallmark and GO biological process terms) and with a second, rank-based algorithm (AUCell, Supplementary Table S9, Figure A2). The two algorithms agreed on the direction of the difference for 7 of 10 sets, including HALLMARK_GLYCOLYSIS (higher in SPP1^+^ TAMs in 47 of 47 samples by both methods), HALLMARK_OXIDATIVE_PHOSPHORYLATION, HALLMARK_FATTY_ACID_METABOLISM, glutamine metabolism and HALLMARK_HYPOXIA. Two composite sets behaved differently, and the reason is informative rather than technical. HALLMARK_CHOLESTEROL_HOMEOSTASIS was higher in SPP1^+^ TAMs, whereas the synthesis genes LDLR, HMGCS1 and SQLE were lower, and the efflux genes ABCA1 and ABCG1 and APOE were higher (Supplementary Table S4); a set that combines synthesis with efflux therefore moves in the opposite direction to its synthesis component, which is the pattern expected if LXRα activation shifts the balance from synthesis toward efflux. Similarly, the GO antigen-processing set aggregates MHC class II structural genes with lysosomal proteases: CTSB, CTSD, CTSL, CTSK and LGMN were all induced (log2FC +0.35 to +2.19), whereas HLA-DRA, HLA-DRB1, HLA-DPA1, HLA-DPB1, HLA-DMA, HLA-DMB, CD74 and CIITA were uniformly reduced. This dissociation between an induced degradation machinery and reduced MHC class II molecules is itself consistent with a defect at the level of presentation rather than of peptide generation, but it also means that composite set scores for these two pathways are method dependent. The metabolic conclusion is therefore based on the directions shared by both gene-set families and both algorithms, and the antigen-presentation statement on the gene-level result.

### 2.7. Transcriptional Regulatory Network of SPP1^+^ TAMs

To reveal the transcriptional regulatory mechanisms associated with this phenotype, the activities of all 271 transcription factors were inferred and the complete ranking was reported (Figure 4C,D, Table 5, Supplementary Table S10). The top five most significantly upregulated activities were POU2F2, SMAD1, NR1H3, HIF1A, and CEBPD.

HIF1A activity was significantly upregulated (ranked 4th), and its target genes BNIP3, SLC2A1, ENO2 and PLOD2 were synchronously upregulated, with the KEGG HIF-1 signaling pathway significantly enriched, collectively supporting the “hypoxia→HIF1A→glycolysis program” pathway. NR1H3 (LXRα) activity ranked 3rd, and LXR activation can inhibit SREBP2-mediated cholesterol synthesis, consistent in direction with the downregulated cholesterol metabolism score and the downregulation of LDLR and SQLE observed in this study, providing a possible transcriptional explanation. PPARG was not a major driving factor in this dataset (rank 105 of 271), so these data do not support PPARG as the core regulator of the lipid phenotype.

### 2.8. Spatial Localization and Microenvironmental Features of SPP1^+^ TAMs

**Figure 5:**
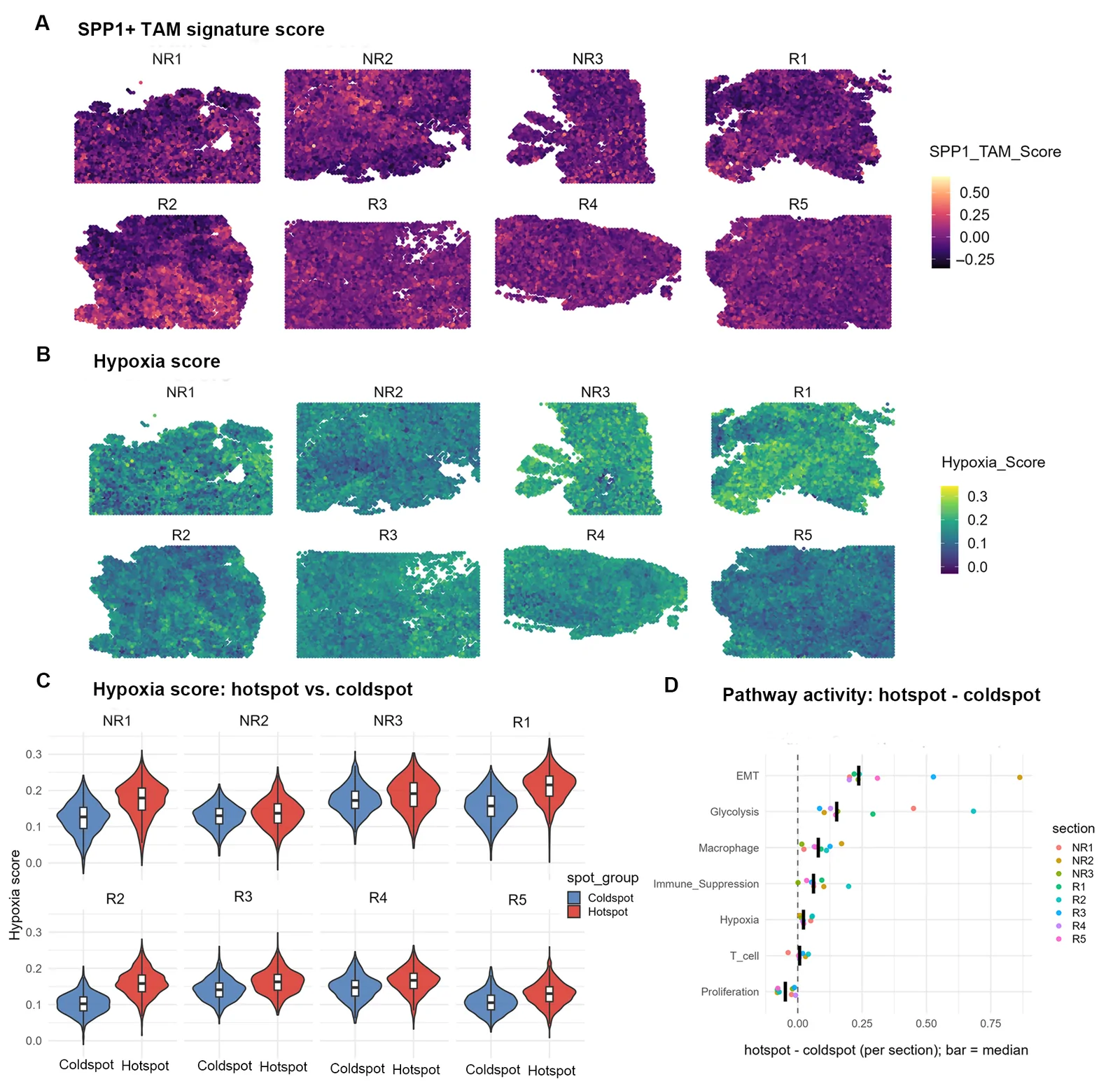
Spatial localization and microenvironmental features of SPP1^+^ TAMs (8 treatment-naïve HGSOC sections). (**A**) Spatial distribution of the SPP1^+^ TAM signature score across the 8 Visium sections (faceted by section; NR1–NR3, non-recurrent tumors; R1–R5, recurrent tumors); (**B**) Spatial distribution of the hypoxia score across the same sections; (**C**) Comparison of hypoxia scores between hotspot and coldspot regions (each accounting for 25% within each section); (**D**) Differences in pathway scores between hotspot and coldspot regions (each point represents one section, with horizontal lines indicating medians; the section was used as the statistical unit, n = 8, Wilcoxon signed-rank test).

**Figure A3:**
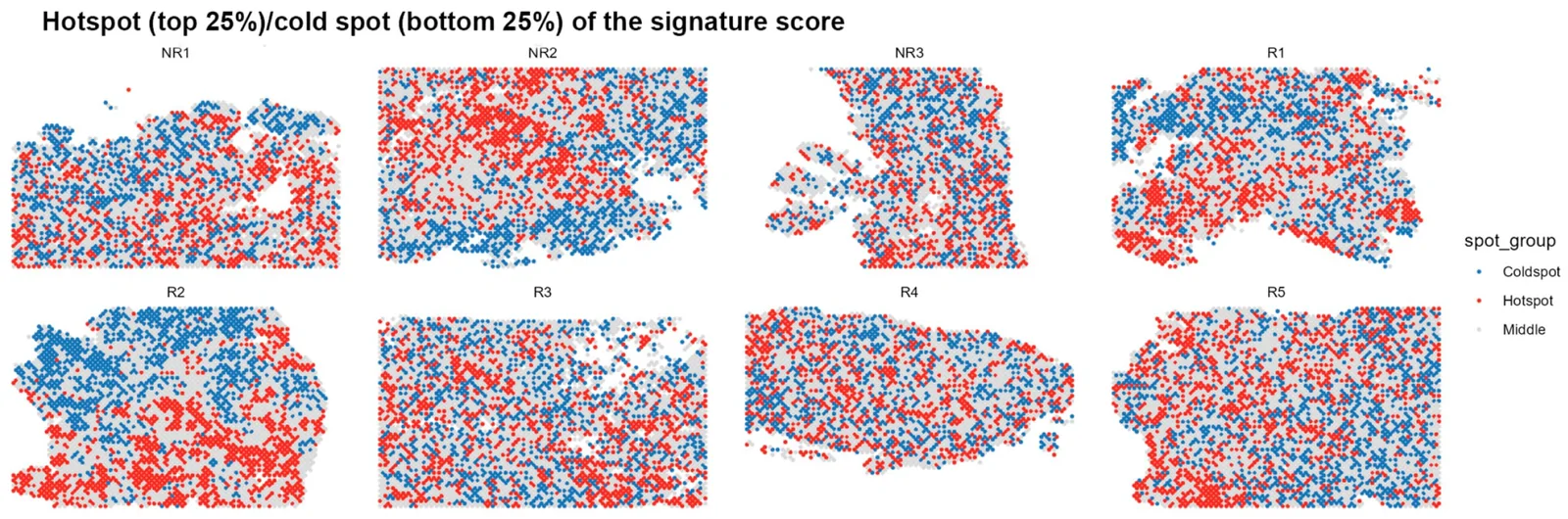
Side-by-side comparison of the SPP1^+^ TAM signature score and the hypoxia score on the same section. Each column pair corresponds to one Visium section (NR1–NR3, non-recurrent; R1–R5, recurrent); the SPP1^+^ TAM signature score is shown in the upper row and the hypoxia score in the lower row, with a common color scale within each pair.

The SPP1^+^ TAM signature score (19 genes, with SPP1 itself excluded, Supplementary Table S11) was mapped onto 8 treatment-naïve HGSOC Visium sections (29,775 spots in total). The score exhibited pronounced heterogeneity across tissue, manifesting as alternating focal high-score regions (hotspots) and low-score regions (coldspots) (Figure 5A). Hotspots and coldspots were defined separately within each section (each accounting for 25%, with 14,894 spots participating in the comparison; the distribution across the 8 sections is shown in Figure 5A, and the two scores are compared side by side in Figure A3.

Notably, the hotspot regions of the SPP1^+^ TAM signature score displayed a good spatial correspondence with the high-value regions of the hypoxia score: in all 8 sections, regions with higher SPP1^+^ TAM signature scores simultaneously exhibited higher hypoxia scores (Figure 5A,B; median Spearman r = 0.344). This pattern was observable in both non-recurrent (NR1–NR3) and recurrent (R1–R5) cases, suggesting a spatial coupling between SPP1^+^ TAMs and the hypoxic microenvironment. With the section as the statistical unit (n = 8), the glycolysis, Epithelial–Mesenchymal Transition (EMT), macrophage, immunosuppression, and hypoxia scores in hotspot regions were elevated in all 8 sections (Figure 5C,D, Table 6), whereas the proliferation score was reduced; the median direction of the T cell score was positive but did not reach statistical significance. Among these, the elevation of the hypoxia score corroborates the upregulated HIF1A activity and HIF-1 pathway enrichment at the single-cell level. The synchronous elevation of EMT and immunosuppression scores suggests that SPP1^+^ TAM-enriched regions also carry enhanced tumor cell invasiveness and an immunosuppressive state.

The SPP1^+^ TAM signature genes were also reproducible in independent differential expression analysis of each section (9–15 signature genes were significantly upregulated in hotspots per section; Supplementary Table S12), indicating that this signature is transferable across independent spatial data.

### 2.9. Relationship Between SPP1^+^ TAM Infiltration and CD8^+^ T Cell Exhaustion

**Figure 6:**
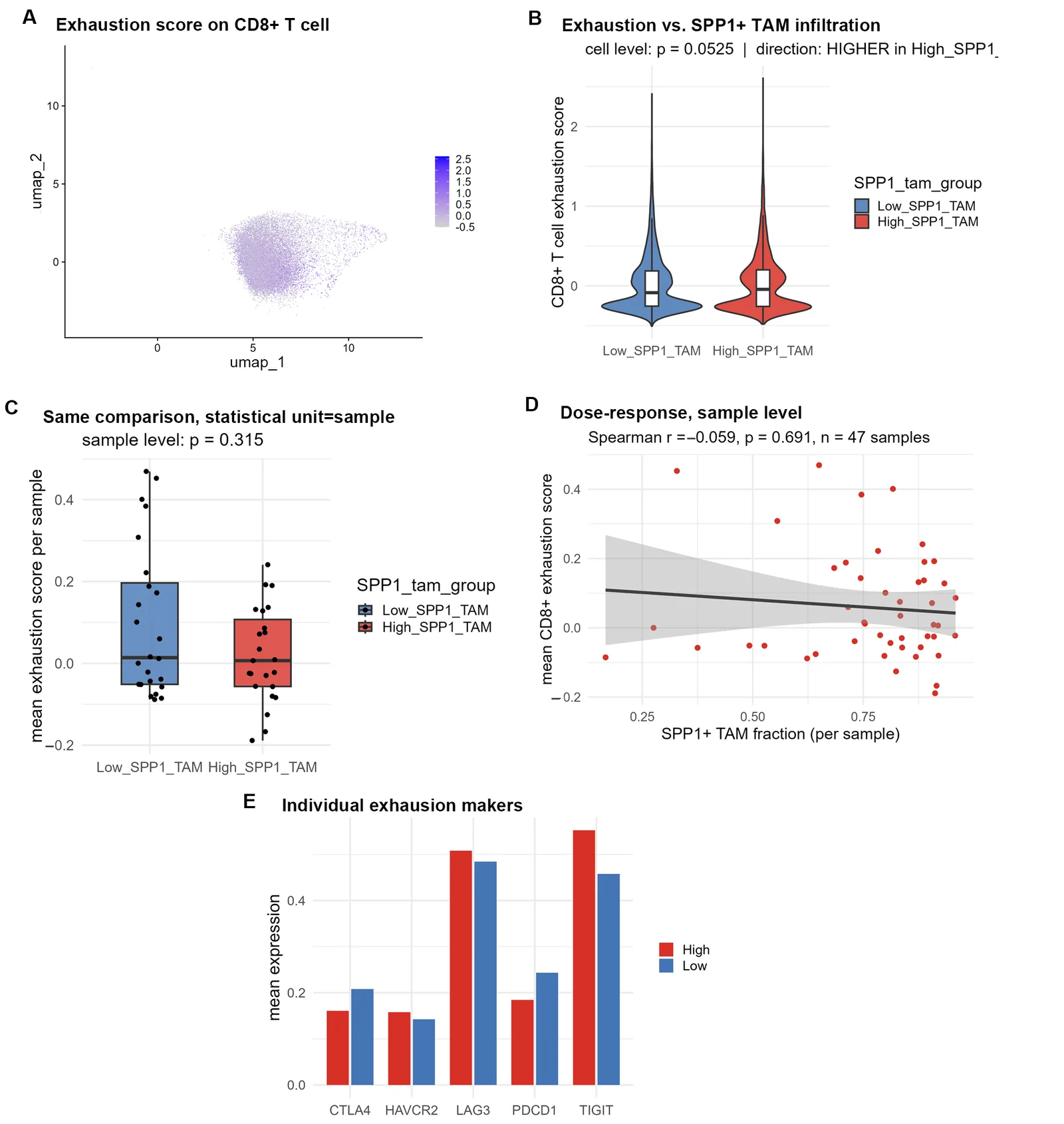
SPP1^+^ TAM infiltration and CD8^+^ T cell exhaustion. (**A**) Distribution of exhaustion scores on the CD8^+^ T cell UMAP; (**B**) cell-level comparison (exploratory, with pseudoreplication); (**C**) sample-level comparison (inferential, 23 vs 24 samples); (**D**) sample-level dose–effect relationship; (**E**) mean expression of individual exhaustion marker genes in the high- versus low-infiltration groups.

A total of 26,459 CD8^+^ T cells were extracted and divided into high- and low-infiltration groups according to the median proportion of SPP1-positive macrophages per sample (23 vs. 24 samples, Figure 6A). Cell-level comparison showed higher exhaustion scores in the high-infiltration group (Figure 6B); this comparison suffers from pseudoreplication and is opposite in direction to the sample-level result, so it cannot be used for inference. At the inferential unit (sample level), no significant difference in CD8^+^ T cell exhaustion scores were observed between the two groups (Figure 6C, Table 7; *p* = 0.315), and the direction was lower in the high-infiltration group. Dose-effect analysis between the sample proportion of SPP1-positive macrophages and the sample mean exhaustion score also showed no association (Figure 6D; Spearman r = −0.059, *p* = 0.691). Gene-by-gene comparison likewise showed no consistent direction (Figure 6E). The conclusion was unchanged when patients rather than samples were used as the unit or when the analysis was restricted to GSE222556 (Supplementary Table S13).

Therefore, in data strictly restricted to treatment-naïve samples, this study demonstrates that SPP1^+^ TAM infiltration is not robustly associated with CD8^+^ T cell exhaustion.

### 2.10. Cell–Cell Communication Network

**Figure 7:**
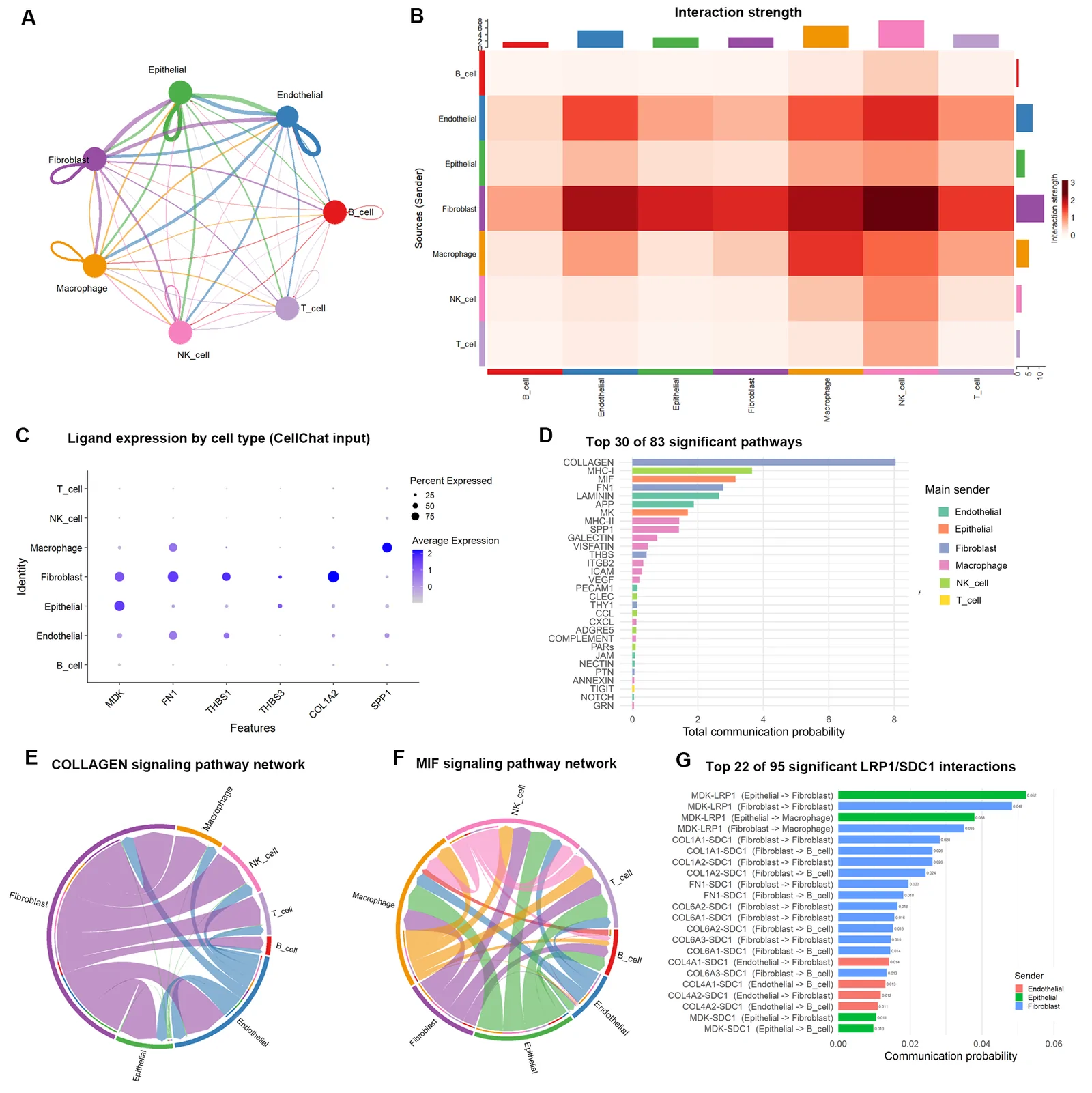
Cell–cell communication network. (**A**) Number of interactions among cell types; (**B**) heatmap of interaction strength; (**C**) expression of key ligands across cell types; (**D**) the 83 significant pathways ranked by overall communication probability; (**E**) chord diagram of the COLLAGEN pathway; (**F**) chord diagram of the MIF pathway; (**G**) top 22 pairs by probability among the 95 LRP1/SDC1 interactions.

To investigate signaling between SPP1^+^ TAMs and tumor cells, CellChat was used to construct a communication network (Figure 7A–C). Among the 83 significant communication pathways, COLLAGEN, MHC-I, MIF, LAMININ, FN1, MK, MHC-II, and SPP1 were all significant (Figure 7D, Supplementary Table S14).

**Figure A4:**
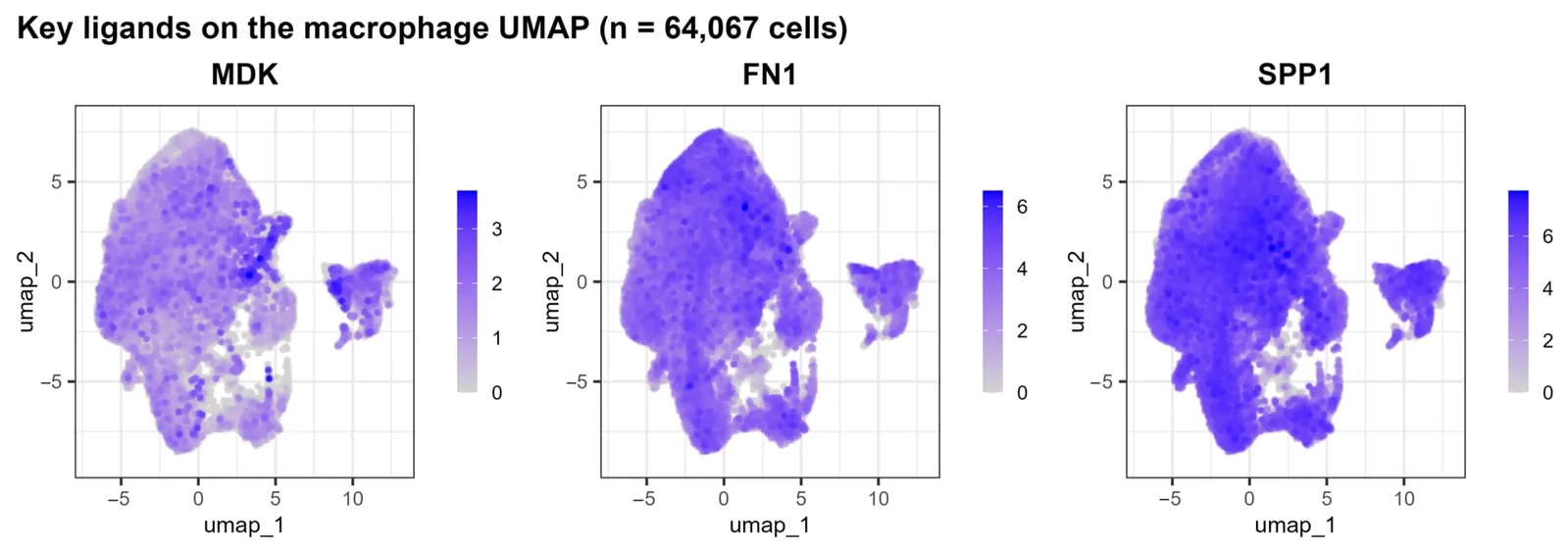
Expression of key ligands on the macrophage UMAP.

The LRP1 and SDC1 receptors together participated in 95 pairs of significant interactions (Figure 7G; complete ranking in Supplementary Table S15), among which the major input in the macrophage direction was MDK–LRP1, whereas COL1A1/COL1A2/COL6A1/A2/A3–SDC1 from fibroblasts constituted a second, continuous group of matrix inputs. MDK was also upregulated in SPP1^+^ TAMs, suggesting that the MK axis may form bidirectional reinforcement loop between tumor and macrophages (Figure A4).

At the pathway level, fibroblast-derived COLLAGEN signaling (total probability 8.04) and epithelial-derived MIF signaling (3.15) ranked highest in strength (Figure 7E,F), suggesting that the microenvironment in which SPP1^+^ TAMs reside is dominated by matrix remodeling signals. LPL is not a ligand in CellChatDB.human, and therefore the direct receptor–ligand action cannot be assessed through this analysis; the effects are more likely mediated indirectly through enzymatic hydrolysis products and microenvironment remodeling. Although the MHC-II pathway was significant in the network, MHC class II gene expression within SPP1^+^ TAMs was reduced, which is compatible with a reduced antigen-presentation program.

### 2.11. Multi-Level Validation of the Hypoxia–SPP1 Association

**Figure 8:**
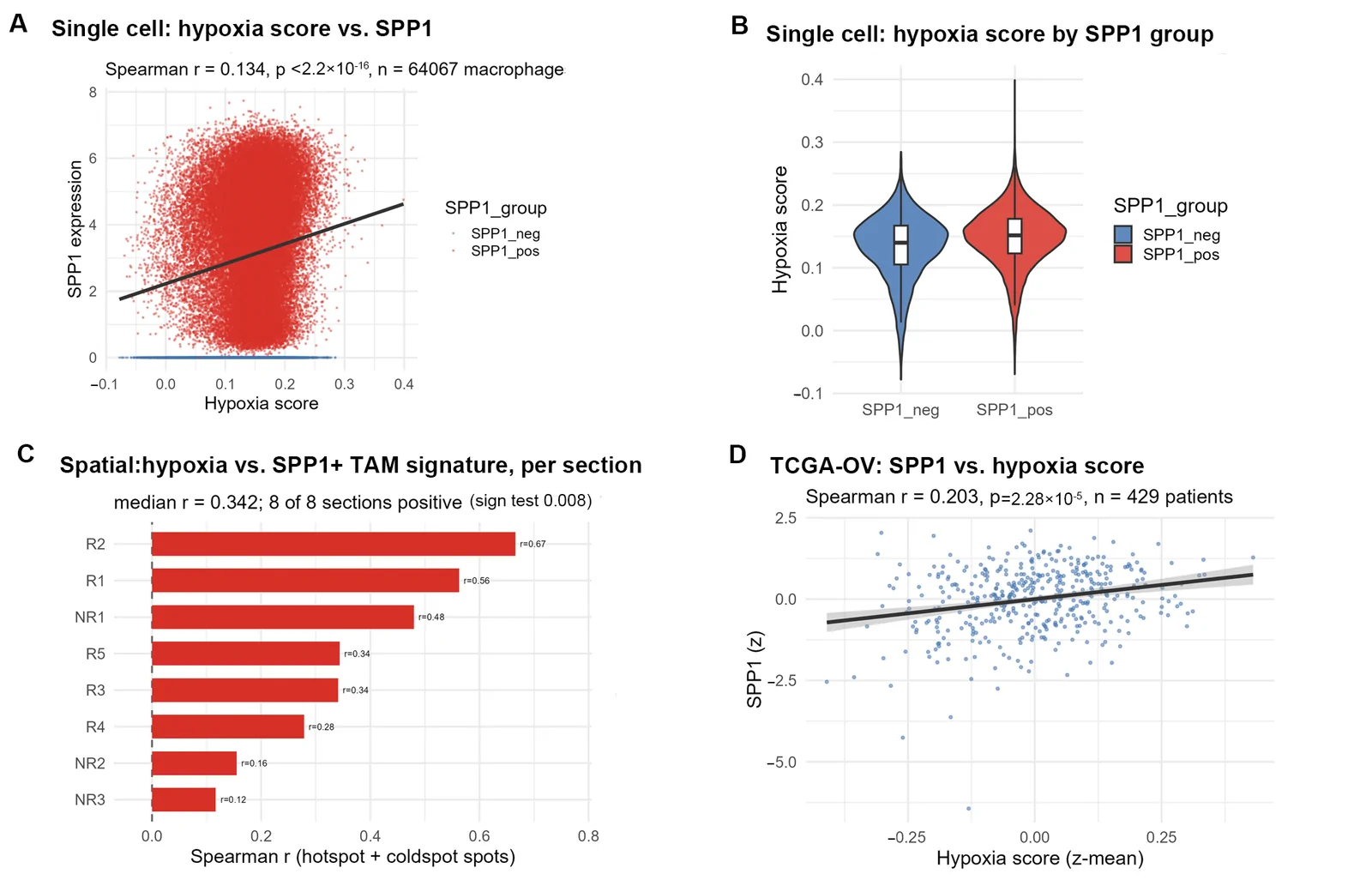
Multi-level validation of the hypoxia–SPP1 association. (**A**) Single-cell: macrophage hypoxia score versus SPP1 expression; (**B**) single-cell: comparison of hypoxia scores between SPP1-positive and SPP1-negative macrophages; (**C**) spatial: correlation between spot-level hypoxia score and SPP1^+^ TAM signature score; (**D**) TCGA-OV cohort (429 cases).

Based on the results of upregulated HIF1A activity and KEGG HIF-1 pathway enrichment, the association between hypoxia and SPP1 was further validated at the single-cell, spatial, and patient levels (Figure 8, Table 8).

Single-cell level: Macrophage hypoxia scores were positively correlated with SPP1 expression (Spearman r = 0.134, *p* < 2.2 × 10^−16^; Figure 8A,B), although cells are not independent replicates. At the sample level, the proportion of SPP1-positive macrophages was positively but not significantly correlated with the mean macrophage hypoxia score (r = 0.234, *p* = 0.113, n = 47); we therefore report this as a consistent trend rather than as a significant association. Spatial level: In the 8 treatment-naïve HGSOC sections, spot-level hypoxia scores were positively correlated with the SPP1^+^ TAM signature score in all 8 sections (Figure 8C; median r = 0.344, sign test *p* = 0.0078); hypoxia scores in hotspot regions were significantly higher than in coldspots (Figure 5C). Patient level: In The Cancer Genome Atlas—Ovarian Cancer (TCGA-OV) cohort (429 cases), SPP1 expression was positively correlated with the hypoxia score (Figure 8D; Spearman r = 0.203, *p* = 2.28 × 10^−5^). The direction of the association was therefore consistent across the three levels, and it reached significance in the spatial and TCGA analyzes but not in the sample-level single-cell analysis; because the data are observational, none of these analyzes establishes a causal role of hypoxia.

Overall, the correlation coefficients at the three levels were consistent in direction and all positive, collectively supporting a positive coupling between hypoxia and SPP1 expression. It should be noted that the observational data in this study can only suggest an association of this axis; whether hypoxia directly drives macrophage polarization toward the SPP1^+^ phenotype still requires functional experimental validation.

### 2.12. In Vivo Validation

**Figure 9:**
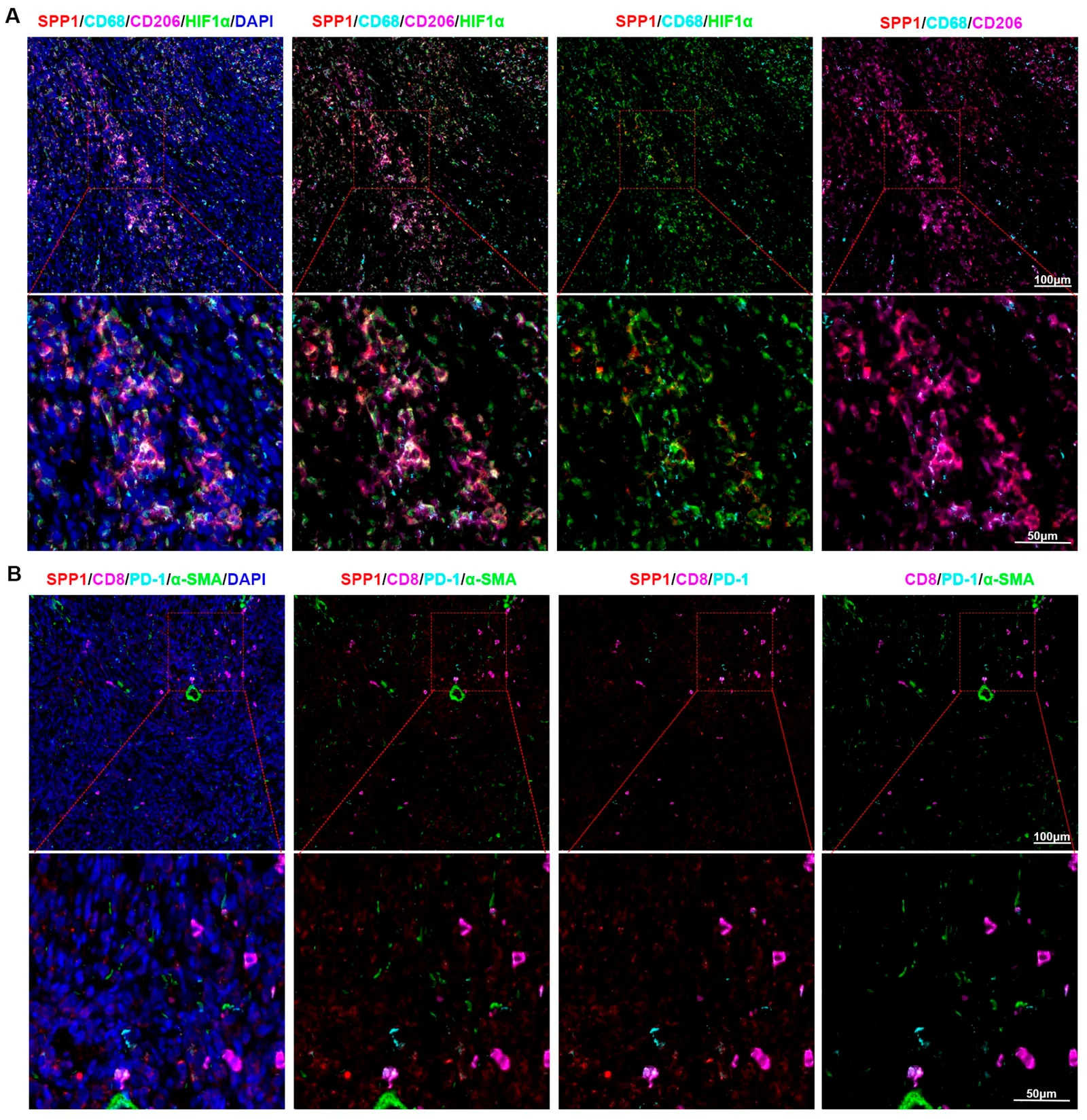
Multiplex immunofluorescence validation of SPP1+ TAMs within hypoxic areas of syngeneic ID8 tumors. (**A**) Representative fields stained for SPP1 (red), CD68 (cyan), CD206 (magenta), HIF1α (green) and DAPI (blue) in subcutaneous ID8 tumors; the upper row shows low-magnification views with the boxed region enlarged below. (**B**) Representative fields stained for SPP1 (red), CD8 (magenta), PD-1 (cyan), α-SMA (green) and DAPI (blue). CD68 marks cells of the macrophage lineage and SPP1 immunoreactivity within CD68^+^ cells identifies SPP1-expressing macrophages; HIF1α-rich and adjacent HIF1α-low areas were imaged on the same section under identical settings. Panels are representative images from six mice and are not a quantitative comparison of hypoxic and non-hypoxic regions.

To examine whether SPP1^+^ macrophages are spatially associated with hypoxic areas in vivo, we used a syngeneic subcutaneous ID8 ovarian tumor model in immunocompetent C57BL/6 mice. Multiplex immunofluorescence was used to detect SPP1, CD68, CD206 and HIF1α on the same sections (Figure 9A). CD68 was used to identify cells of the macrophage lineage; SPP1 immunoreactivity within CD68^+^ cells identified SPP1-expressing macrophages. Within each tumor, HIF1α-rich areas and adjacent HIF1α-low areas were identified on the same section. In the HIF1α-rich areas examined, SPP1 immunoreactivity was detectable in a subset of CD68^+^ macrophages, and CD206^+^ cells were present in the same field (Figure 9A); we describe this as spatial co-localization of SPP1-expressing macrophages with hypoxic areas rather than as an enrichment, because the number of SPP1^+^CD68^+^ cells per field was limited. Consistent with the sample-level exhaustion analysis, CD8^+^PD-1^+^ T cells were sparse and did not form aggregates in the areas examined (Figure 9B). These observations are descriptive: they show that SPP1-expressing macrophages can be found within hypoxic regions of an ID8 tumor, but they do not establish that hypoxia or HIF1A drives the SPP1^+^ state, and they do not provide a quantitative hypoxic-versus-non-hypoxic comparison.

## 3. Discussion

This study integrated 398,665 cells from 47 treatment-naïve tumor samples of 47 patients across three independent datasets to construct a single-cell atlas of the treatment-naïve ovarian cancer microenvironment in which every macrophage analyzed is tumor-derived and every patient contributes once. At the methodological level, conventional fixed-threshold quality control introduced a systematic bias against datasets with high annotation depth: across the complete deposited series of GSE184880, fixed thresholds retained only 63.8% of cells, whereas the adaptive standard retained 79.4%. Fixed mitochondrial percentage and gene-count thresholds cannot accommodate the heterogeneity in transcriptomic features introduced by different dissociation protocols, library chemistries and sequencing depths, and can therefore remove transcriptionally active viable cells or retain compromised ones. The adaptive strategy used here sets filtering boundaries from sample-specific distributions, which balances signal preservation against noise removal.

Macrophages accounted for 16.62% of all cells and formed the third largest population after T cells and epithelial cells, consistent with the recognition of ovarian cancer as a macrophage-rich tumor. Two observations about the SPP1 definition are central. First, SPP1 was not restricted to a discrete subpopulation: the proportion of SPP1^+^ cells ranged from 29.0% (subpopulation 15) to 100.0% (subpopulation 16) across the 18 macrophage subpopulations, and the positivity rate changed with the cut-off (82.0% at SPP1 > 0, 76.1% at SPP1 > 1, 67.2% at SPP1 > 2). Hou et al. defined SPP1^+^ TAMs as an independent subpopulation and linked them to T cell exhaustion through SPP1–CD44 [13]; our data suggest instead that SPP1 expression is closer to a cross-cluster functional state, which shifts the therapeutic question from how to deplete this subpopulation to how to interrupt the upstream program that maintains it. Second, this interpretation is not an artefact of the threshold: when the comparison was made within each sample between SPP1-high and SPP1-low macrophages, the transcriptional and metabolic differences were reproduced. Fan et al. similarly described SPP1^+^ macrophages co-localising with cancer stem cells in hypoxic regions of hepatocellular carcinoma under HIF1A regulation [16], suggesting that SPP1 expression is a general macrophage response to hypoxic stress.

The metabolic characterization deviates from the classical M1/M2 framework. SPP1^+^ TAMs showed higher glycolysis, fatty acid synthesis and glutaminolysis scores and a lower cholesterol synthesis score, without suppression of fatty acid oxidation or oxidative phosphorylation. Dussold et al. argued that the classical pairing of glycolysis with M1 and oxidative phosphorylation with M2 likely underestimates the diversity of in vivo states [17], and the SPP1^+^ phenotype described here is an example of such a hybrid state. The cholesterol result deserves comment: LDLR and SQLE were downregulated, the cholesterol synthesis score was reduced, and NR1H3 (LXRα) activity ranked third. Hoppstädter et al. also reported downregulation of cholesterol biosynthesis genes and upregulation of cholesterol efflux transporters in lung cancer TAMs [18]. One explanation is that LXRα activation in the tumor microenvironment inhibits SREBP2-dependent cholesterol synthesis while promoting efflux, although cholesterol content and flux were not measured. PPARG was not a driver in this dataset [19,20]; the discrepancy with studies in other tumors may reflect cancer-type and microenvironment specificity, including the distinctive lipid composition of the ovarian peritoneal compartment. All of these statements are transcriptional and await protein-level and flux-level confirmation.

Our cross-level analysis found the same positive direction between hypoxia and SPP1 at the single-cell, spatial and TCGA levels, although the sample-level single-cell correlation did not reach significance (*p* = 0.113) and the strongest evidence therefore comes from the spatial (all 8 sections positive) and TCGA analyzes. HIF1A activity was significantly upregulated (rank 4), its target genes BNIP3/SLC2A1/ENO2/PLOD2 were synchronously upregulated, and the KEGG HIF-1 pathway was enriched. However, observational data cannot establish causality, and HIF1A activity may merely accompany the hypoxic niche in which SPP1^+^ TAMs reside; functional experiments are indispensable.

Our sample-level analysis did not find a robust association between SPP1^+^ TAM infiltration and CD8^+^ T cell exhaustion (*p* = 0.315), a negative result that contrasts with Hou et al. [13]. The analytical unit is decisive: Hou et al. may have assessed the association at the cell level, whereas we used the sample as the inferential unit and confirmed the absence of an association when patients were used as the unit (Table S17). In treatment-naïve samples the association may be diluted by tumor burden, ascites volume and anatomical site, and the functional coupling between SPP1^+^ TAMs and T cell exhaustion may not yet be established. The inflamed but not immunologically effective framework of Neri et al. is compatible with exhaustion being the result of several suppressive mechanisms rather than of SPP1^+^ TAMs alone [21]. The negative result does not diminish the pathological significance of SPP1^+^ TAMs; it suggests that their immunosuppressive action may be spatial rather than systemic.

CellChat analysis revealed the communication network features of SPP1^+^ TAMs: fibroblast-derived COLLAGEN signaling and epithelial-derived MIF signaling ranked highest in strength, with the MDK–LRP1 axis constituting the major input in the macrophage direction, suggesting that the functions of SPP1^+^ TAMs may be more regulated by the stromal microenvironment than by direct tumor cell signals. The MHC-II pathway was significant in the communication network while the MHC class II genes and CIITA were downregulated within SPP1^+^ TAMs; this is compatible with, but not proof of, a reduced capacity to present antigen. It should be noted that LPL is not a ligand in CellChatDB.human and that its effects are more likely mediated indirectly through enzymatic hydrolysis products and microenvironment remodeling, indicating that non-canonical functions of metabolic enzymes require metabolomic or functional approaches for resolution.

Taken together, these data define SPP1^+^ TAMs in treatment-naïve HGSOC as a hypoxia-associated functional state with a distinct transcriptional metabolic and antigen-presentation profile, and they identify the questions that must be answered experimentally before this state can be treated as a therapeutic target. The limitations of the evidence are set out in the Section 4.

## 4. Limitations

This study has several limitations. First, all molecular findings are derived from transcriptomic data: the metabolic scores are transcriptional signatures rather than measurements of metabolic flux; the downregulation of HLA-DRA, HLA-DRB1, CD74, and CIITA reflects only a reduced MHC class II transcriptional program rather than impaired antigen presentation function itself (surface MHC class II protein, peptide loading, and functional T cell stimulation were not measured); and the reduction in cholesterol synthesis genes and the increase in glycolysis genes have not been validated at the protein or metabolite level. Therefore, the conclusions regarding metabolic reprogramming and antigen presentation should be regarded as hypothesis-generating. In addition, pathway-level composite scores are sensitive to gene-set composition (e.g., when synthesis genes are combined with efflux genes, or MHC class II structural genes with lysosomal proteases, the score may move in the opposite direction to its functionally relevant members); therefore, Section 2.6 reports both gene-level results and set scores. Second, the study is an observational design and cannot establish causality. The positive correlation between hypoxia and SPP1 expression was consistent at the single-cell, spatial, and TCGA levels, and HIF1A activity and its target genes were also upregulated, but HIF1A activity may be a consequence rather than a cause of the hypoxic microenvironment in which SPP1^+^ TAMs reside. No HIF1A perturbation (deletion, stabilization, or pharmacological inhibition) was performed, and the in vivo experiment is merely descriptive, showing spatial co-localization of SPP1-expressing macrophages with HIF1α-rich areas in one syngeneic model without an untreated control group. Direct tests such as HIF1A loss- and gain-of-function, SPP1 promoter occupancy, and in vivo SPP1 blockade remain to be performed. Third, SPP1 positivity is threshold dependent; although the principal findings were reproduced by within-sample high-versus-low comparisons, absolute positivity rates should not be compared across studies. Fourth, residual batch structure was still detectable in a small number of rare clusters, the macrophage compartment was necessarily collapsed into two groups for differential analysis, and the cell–cell communication analysis was performed on a stratified subsample. Finally, the negative result for CD8^+^ T cell exhaustion does not exclude a functional interaction that would be detectable in larger samples, paired spatial and proteomic measurements, or perturbation experiments. Future work should integrate the directions outlined above.

## 5. Materials and Methods

### 5.1. Data Sources and Sample Inclusion/Exclusion

Three publicly available ovarian cancer single-cell transcriptomic datasets were obtained from the Gene Expression Omnibus (GEO) database. Only treatment-naïve tumor samples were included: post-treatment samples were excluded, non-malignant ovarian samples were excluded, and when a patient contributed several lesions only one lesion was retained, so that the statistical unit (sample) and the biological unit (patient) coincide. The dataset characteristics were as follows:

GSE184880: 12 samples profiled; the 5 normal ovarian samples (GSM5599220–GSM5599224, GEO tissue type “Normal ovarian tissue”) were excluded and the 7 treatment-naïve HGSOC samples (GSM5599225–GSM5599231) were retained;

GSE222556: 28 post-treatment samples excluded and 39 pre-treatment samples available; of these, 34 samples from 34 patients were retained by keeping the primary lesion when a patient contributed both a primary and a metastatic lesion (P04, P06, P08, P15) and by keeping a single biopsy when two replicates of the same primary lesion were deposited (P04_P1-1 and P04_P1-2);

GSE241221: 4 post-treatment samples excluded and the 6 untreated patient samples retained.

For spatial transcriptomics, GSE263920 was used, comprising 10× Visium sections from 8 treatment-naïve HGSOC patients who underwent cytoreductive surgery (NR1–NR3: non-recurrent; R1–R5: recurrent).

The expression matrix and clinical information of the TCGA-OV cohort were obtained from the Genomic Data Commons data portal for external validation.

### 5.2. Quality Control

Per-sample adaptive quality control was applied in this study: for each sample, the median and median absolute deviation (MAD) of log_10_(detected gene count) and log_10_(total UMI count) were calculated separately, and cells deviating from the median by ±3 MAD were removed (equivalent to the isOutlier strategy in scater), with absolute lower limits of detected gene count ≥200 and mitochondrial gene proportion <20% uniformly applied. In parallel, the retention counts under conventional fixed thresholds (gene count 200–5000, UMI 500–20,000, mitochondrial proportion <20%) were calculated for comparison.

### 5.3. Gene Lineage Coordination, Integration, and Clustering

The intersection of genes across the three datasets (17,356 genes) was taken to ensure a consistent feature space. Seurat v5 was used to perform LogNormalize normalization (scale factor = 10,000), and 2000 highly variable genes were selected using the vst method. Principal Component Analysis (PCA) dimensionality reduction was performed with 50 principal components, and Harmony was employed to correct batch effects using dataset as the batch variable. The top 30 principal components were used for UMAP dimensionality reduction (min.dist = 0.3, n.neighbors = 30) and Louvain clustering (resolution = 0.8), yielding a total of 55 clusters.

### 5.4. Cell Type Annotation

Clusters were annotated based on classical marker genes: EPCAM/KRT8/KRT18/PAX8 (epithelial cells), CD3D/CD3E/CD2 (T cells), CD68/CD163/CD14/AIF1 (macrophages), MS4A1/CD79A/CD79B (B cells), NKG7/GNLY/KLRD1 (NK cells), COL1A1/COL1A2/DCN (fibroblasts), and PECAM1/VWF/CDH5 (endothelial cells).

### 5.5. Definition of SPP1^+^ TAMs and Threshold Sensitivity Analysis

All macrophages were extracted and re-clustered without using SPP1, yielding 18 subpopulations. Cells were divided into positive (normalized SPP1 > 0) and negative (SPP1 = 0) groups; the threshold was varied in the sensitivity analysis.

### 5.6. Differential Expression Analysis

Cell-level differential expression was performed using FindMarkers (Wilcoxon rank-sum test, min.pct = 0.1, logfc.threshold = 0), with significance defined as |log2FC| > 0.5 and *p*_adj_ < 0.05. To avoid the pseudoreplication problem of cell-level testing, count matrices were additionally aggregated by “dataset × sample × group” as units, and sample-level differential expression analysis was performed using edgeR, with the reproduction proportion of the cell-level Top-20 upregulated genes in the sample-level analysis reported.

### 5.7. Functional Enrichment and Gene Set Enrichment Analysis

Using all tested genes (17,356) as background, clusterProfiler was used to perform GO enrichment analysis on upregulated genes (482 genes, all successfully mapped to Entrez IDs), with biological process (BP), cellular component (CC), and molecular function (MF) ontologies reported separately, corrected by the Benjamini–Hochberg method, with *p*_adj_ < 0.05 and q < 0.2. KEGG pathway enrichment was performed using enrichKEGG (KEGG REST API). In addition, to utilize the ranking information of all genes rather than only significant genes, GO BP gene sets (4079 sets, gene count 10–500) were constructed offline based on org.Hs.eg.db and GO.db, and all tested genes were ranked by log2FC followed by gene set enrichment analysis using fgsea (10,000 permutations), with normalized enrichment score and False Discovery Rate (FDR) reported.

### 5.8. Metabolic Pathway Scoring

Six metabolic pathways were scored with AddModuleScore. The gene sets were defined a priori and kept deliberately small, so that each score reflects a single enzymatic step or a small reaction chain rather than a whole pathway: glycolysis (HK2, PFKP, PFKM, ALDOA, GAPDH, PKM, LDHA), fatty acid oxidation (CPT1A, CPT2, ACADS, ACADM, ACADL, HADHA, HADHB), fatty acid synthesis (FASN, ACACA, ACACB, SCD, ELOVL6), cholesterol synthesis (LDLR, HMGCR, HMGCS1, SQLE, ACAT2), oxidative phosphorylation (NDUFB8, SDHB, UQCRC2, COX4I1, ATP5A1) and glutaminolysis (SLC1A5, GLS, GLUD1, GOT1). The rationale was to capture the specific enzymatic modules that were transcriptionally regulated in the differential expression analysis rather than to estimate pathway flux. Two limitations follow from this design and are stated explicitly: the scores are transcriptomic signatures and not measurements of metabolic activity, and small sets are sensitive to the behavior of individual genes. To control for both, all six pathways were rescored with established curated gene sets (MSigDB Hallmark v2024.1 for glycolysis, oxidative phosphorylation, cholesterol homeostasis, fatty acid metabolism and hypoxia; GO biological process terms GO:0009062, GO:0006633, GO:0006541 and GO:0006695 for fatty acid catabolism, fatty acid biosynthesis, glutamine metabolism and cholesterol biosynthesis) and with a second, rank-based scoring algorithm (AUCell), and the direction of the difference was compared across methods.

### 5.9. Transcription Factor (TF) Activity Inference

The dorothea database (confidence levels A/B) was used in conjunction with the run_mlm method of decoupleR to infer the activities of all 271 transcription factors, and the complete ranking was reported rather than selectively reported (Supplementary Table S8).

### 5.10. Spatial Transcriptomics Analysis

Eight treatment-naïve HGSOC Visium sections from GSE263920 were used (30,209 raw spots, 29,775 spots after quality control). The SPP1^+^ TAM signature score was composed of the Top-19 upregulated genes from cell-level differential expression, with SPP1 itself excluded to avoid the circular reasoning of “defining hotspots by the signature and then claiming signature enrichment” (signature gene list in Supplementary Table S11). Hotspots (Top 25% of signature score within each section) and coldspots (Bottom 25%) were defined separately within each section to avoid artifacts caused by inter-section differences in staining and sequencing depth; subsequently, with the section as the statistical unit (n = 8), a Wilcoxon signed-rank test was performed on the “hotspot − coldspot” score difference for each section. The overlap between the signature and hotspot-upregulated genes in each section was also calculated section by section.

### 5.11. Multi-Level Validation of the Hypoxia–SPP1 Association

The hypoxia gene set was derived from genes annotated under GO:0001666 (response to hypoxia) and GO:0071456 (cellular response to hypoxia) in org.Hs.eg.db (278 genes; the actual list used and its source are provided in Supplementary Table S16) to ensure reproducibility in an offline environment. Validation was performed at three levels: (1) at the single-cell level, hypoxia scores of macrophages were calculated, and cell-level (exploratory) and sample-level (inferential) correlations were reported; (2) at the spatial level, hypoxia scores and SPP1^+^ TAM signature scores were calculated for each spot, Spearman correlations were calculated by section, and the 8 sections were summarized using a sign test; (3) at the patient level, the TCGA-OV expression matrix (gene columns as versioned Ensembl IDs, used after mapping and deduplication) was z-standardized by gene, and hypoxia scores (277 genes available) were calculated and correlated with SPP1 expression using Spearman correlation.

### 5.12. CD8^+^ T Cell Exhaustion Analysis

CD8A^+^ T cells (26,459 cells) were extracted, and exhaustion scores were calculated from PDCD1, LAG3, HAVCR2, TIGIT and CTLA4. Samples were divided into high- and low-infiltration groups according to the median proportion of SPP1-positive macro-phages per sample (23 vs. 24 samples). Cell-level (exploratory) and sample-level (inferential) Wilcoxon tests were performed separately, and the dose–effect relationship between the sample proportion of SPP1-positive macrophages and the sample mean exhaustion score was calculated.

### 5.13. Cell–Cell Communication Analysis

CellChat was used to construct the cell–cell communication network. Because the computational load of permutation testing grows with the square of the cell number, the analysis was performed on a documented stratified subsample (a maximum of 300 cells per dataset × cell type stratum, totalling 6216 cells; composition in Table S17). Communication probabilities were calculated with the triMean method, with filter-Communication requiring min.cells = 10 and permutation testing with n = 1000.

### 5.14. Construction of a Syngeneic Subcutaneous Ovarian Tumor Model

Female C57BL/6 mice aged 4–6 weeks (n = 6) were used. ID8 mouse ovarian surface epithelial carcinoma cells (1 × 10^7^ per mouse, from the same preparation and the same passage) were inoculated subcutaneously into the flank of every animal, so that all mice received an identical inoculum. No untreated or vehicle-treated control group was included: the experiment was designed as a descriptive, within-tumor comparison, and all comparisons were made on the same section between HIF1α-rich areas and adjacent HIF1α-low areas. Tumors were harvested when they reached the predetermined experimental volume (approximately two months after inoculation). ID8 cells are of C57BL/6 origin and were implanted into immunocompetent C57BL/6 recipients, so this is a syngeneic allograft model. Mice were anesthetized by intraperitoneal injection of sodium pentobarbital, followed by cervical dislocation before tumor excision. All animal experiments followed the Guide for the Care and Use of Laboratory Animals of the National Institutes of Health and were approved by the Experimental Animal Ethics Committee of Renmin Hospital of Wuhan University (approval number WDRM20230201A).

### 5.15. Multiplex Immunohistochemistry Staining

Paraffin sections of tumor tissue with a thickness of 5 μm were subjected to dewaxing, hydration, antigen retrieval, and blocking of endogenous peroxidase activity, followed by detection of the following target proteins: SPP1 + CD68 (Servicebio, Wuhan, China, Cat. No. GB113109) + CD206 (Servicebio, Cat. No. GB113497) + HIF1α; SPP1 + CD8 (Abcam, Cat. No. ab237709) + PD-1 (Abcam, Cambridge, UK, Cat. No. ab309363) + α-SMA (Boster, Wuhan, China, Cat. No. BM0002). Each round of staining proceeded as follows: primary antibody incubation at 4 °C overnight, followed by addition of horseradish peroxidase (HRP)-labeled secondary antibody; after each round of staining, antigen stripping was performed to remove residual antibodies and avoid cross-reactivity. Nuclei were counterstained with DAPI, and images were acquired after mounting with anti-fluorescence quenching mounting medium (Servicebio).

### 5.16. Robustness to the Definition of SPP1 Positivity

The reference definition of SPP1 positivity (normalized expression > 0) was challenged with two definitions that do not depend on absolute expression. Within every sample with at least 60 macrophages, cells were ranked by SPP1 expression and split into tertiles (high = top third, low = bottom third, middle third excluded) or into halves at the sample median. Differential expression (Wilcoxon rank-sum, |log2FC| > 0.5, *p*_adj_ < 0.05), the six metabolic scores and the MHC class II panel were recomputed for each definition and compared with the reference list; concordance was quantified as the proportion of reference differentially expressed gene recovered and as the sign agreement of the effect sizes. Because both members of each comparison come from the same sample, this design also removes any sample-level difference in sequencing depth or cell composition.

### 5.17. Sensitivity Analyzes of the Sample Restrictions

The consequences of the sample restrictions were quantified in three ways. (i) The number of macrophages contributed by the five non-malignant ovarian samples and their SPP1 positivity rate were calculated from the unrestricted atlas. (ii) In GSE222556, the SPP1 positivity rate of primary and metastatic lesions was compared at the sample level (Wilcoxon rank-sum) and, in the four patients who contributed both lesion types, within patient (paired Wilcoxon signed-rank test). (iii) The association between SPP1^+^ TAM infiltration and CD8^+^ T cell exhaustion was recomputed with the sample, the patient and the treatment-naïve subset as the unit of analysis, and the confidence interval of the correlation was obtained by bootstrapping patients rather than samples (2000 resamples).

### 5.18. Curated Gene Sets and Independent Pathway Scoring

Curated gene sets were read from the MSigDB Hallmark collection (h.all.v2024.1.Hs.symbols.gmt) and from GO biological process terms resolved offline through org.Hs.eg.db. Each set was scored by Add-ModuleScore and, independently, by AUCell, a rank-based method that does not depend on the mean-variance relationship of the normalized counts. For AUCell, the ranking matrix was restricted to the union of the curated sets and the 2000 variable genes, and the area under the recovery curve was calculated with aucMaxRank set to the top 5% of the ranked genes. Between-group differences were tested at the sample level with paired Wilcoxon signed-rank tests, and the agreement between the two scoring algorithms was assessed by the sign of the median paired difference.

### 5.19. Statistical Analysis

All analyses were completed in R 4.6.1. Wilcoxon rank-sum tests were used for two-group comparisons, and Spearman correlation coefficients were used for correlation analysis; cross-section consistency was tested using the Wilcoxon signed-rank test and the sign test. The Benjamini–Hochberg method was used to control the false discovery rate in analyses involving multiple comparisons. Significance thresholds: *p* < 0.05, *p* < 0.01, *p* < 0.001. The statistical unit is explicitly indicated in each result: cell-level, spot-level, and within-section comparisons serve only as exploratory evidence due to pseudoreplication, and inferential conclusions are based solely on sample-level, section-level, or patient-level tests.

## 6. Conclusions

This study characterized the molecular features and functional phenotype of SPP1^+^ TAMs in treatment-naïve ovarian cancer at the single-cell, tissue-spatial and patient levels. The main conclusions are as follows: (1) SPP1 marks a continuous functional state of macrophages rather than a discrete subpopulation—SPP1^+^ cells were distributed across all 18 macrophage subpopulations, the positivity rate depended on the expression threshold (82.0% at SPP1 > 0 and 67.2% at SPP1 > 2) and on sequencing depth, and the molecular differences were reproduced with within-sample high-versus-low comparisons; (2) at the transcriptional level this state is accompanied by increased glycolysis, increased lipid uptake and synthesis gene expression and reduced cholesterol synthesis gene expression, a combination that does not correspond to the classical M1/M2 dichotomy and that requires protein-level and flux-level confirmation before it can be described as a metabolic reprogramming; (3) HIF1A activity and its downstream program were upregulated, and hypoxia and SPP1 were positively associated at the single-cell, spatial and TCGA levels in all 8 sections, which identifies the hypoxia–HIF1A axis as a candidate upstream regulator; this remains an association, because HIF1A perturbation and direct promoter assays were not performed; (4) the same state shows coordinated downregulation of MHC class II genes and CIITA, consistent with a reduced antigen-presentation program; because surface protein and functional presentation were not measured, impaired antigen presentation remains a hypothesis to be tested rather than a demonstrated immune-evasion mechanism; and (5) after restricting the analysis to treatment-naïve samples, no robust association was observed between SPP1^+^ TAM infiltration and CD8^+^ T cell exhaustion, suggesting that previous conclusions may have been confounded by treatment exposure.

## Figures and Tables

**Table 1:** Datasets included and quality control results.

Dataset	Sample Type	Samples (Patients)	Platform	Cells Before QC	Cells After QC	Retention (%)	Median Genes/Cell	Median mt (%)
GSE 184880	HGSOC	7 (7)	10× Genomics	38,775	27,035	69.7	2016	6.07
GSE 222556	HGSOC	34 (34)	10× Genomics	340,170	338,749	99.6	2409.75	4.85
GSE 241221	HGSOC	6 (6)	10× Genomics	19,720	19,720	100	1976.5	4.37

**Table 2:** Cell type composition of the ovarian cancer single-cell atlas.

Cell Type	n Cells	Percentage (%)
T cell	94,463	24.50
Epithelial	87,474	22.69
Fibroblast	65,039	16.87
Macrophage	64,067	16.62
NK cell	44,638	11.58
B cell	17,193	4.46
Endothelial	12,630	3.28

**Table 3:** Definition of SPP1-positive macrophages and threshold sensitivity.

Threshold	n SPP1^+^	n SPP1^−^	SPP1^+^ (%)	Median SPP1 in Positive Cells
SPP1 > 0	52,512	11,555	81.96	4.029
SPP1 > 1	48,738	15,329	76.07	4.186
SPP1 > 2	43,077	20,990	67.24	4.404

**Table 4:** Metabolic pathway scores (cell-level and sample-level).

Pathway	Mean SPP1^+^	Mean SPP1^−^	Δ Cell	Samples Higher in SPP1^+^	Mean of Sample Means SPP1^+^	Mean of Sample Means SPP1^−^	Δ Sample	*p* (Sample)
Cholesterol	−0.0027	0.0258	−0.0285	6/47	−0.0046	0.0169	−0.0215	3.52 × 10^−7^
FAO	0.0147	0.0149	−2 × 10^−4^	29/47	0.0139	0.0074	0.0065	0.057
FA_Synthesis	0.0124	−0.0214	0.0338	47/47	0.0143	−0.0219	0.0362	1.42 × 10^−14^
Glutaminolysis	0.0247	0.0071	0.0176	31/47	0.0224	0.0094	0.0131	0.0216
Glycolysis	0.303	0.082	0.221	47/47	0.3498	0.1165	0.2333	1.42 × 10^−14^
OXPHOS	0.038	0.0358	0.0023	27/47	0.0527	0.0496	0.003	0.363

**Table 5:** Ranking of transcription factor activities.

Rank	TF	Mean Activity SPP1^+^	Mean Activity SPP1^−^	Δ Activity
1	POU2F2	2.5531	0.7045	1.8486
2	SMAD1	2.0617	0.6293	1.4324
3	NR1H3	1.3272	0.2558	1.0713
4	HIF1A	2.9764	2.03	0.9464
5	CEBPD	1.8716	1.1353	0.7363
6	STAT2	2.1507	1.4797	0.671
7	EGR1	0.4559	−0.1792	0.6351
8	JUN	0.4368	−0.1546	0.5914
9	POU2F1	1.0568	0.4797	0.577
10	IRF9	1.6604	1.0903	0.5701
11	FOXL2	2.1214	1.6153	0.5061
12	CEBPA	0.0993	−0.3892	0.4885
13	ERG	1.9689	1.4817	0.4871
14	MYCN	0.1057	−0.3685	0.4742
15	TBP	1.4802	1.006	0.4742
21	REL	1.8312	1.4875	0.3437
31	MAF	1.8704	1.6011	0.2692
101	BACH1	0.2827	0.2614	0.0213
105	PPARG	1.9655	1.9513	0.0141
109	KLF5	−0.4765	−0.4838	0.0073

**Table 6:** Comparison of pathway scores between spatial hotspots and coldspots.

Pathway	Sections	Sections Positive	Median Δ	Min Δ	Max Δ	Direction	*p* (Signed Rank)
EMT	8	8	0.237	0.2002	0.8631	higher in hotspot	0.00781
Glycolysis	8	8	0.1513	0.0848	0.6831	higher in hotspot	0.00781
Macrophage	8	8	0.0799	0.0157	0.1702	higher in hotspot	0.00781
Immune_Suppression	8	8	0.0614	0.001	0.1977	higher in hotspot	0.00781
Hypoxia	8	8	0.0222	0.0084	0.0565	higher in hotspot	0.00781
T_cell	8	7	0.0072	−0.0376	0.0411	higher in hotspot	0.148
Proliferation	8	0	−0.0483	−0.0794	−0.0086	lower in hotspot	0.00781

**Table 7:** CD8^+^ T cell exhaustion and SPP1^+^ TAM infiltration.

Comparison	Statistical Unit	n High	n Low	Mean High	Mean Low	Direction	Spearman r	n Samples	*p* Value
cell level (pseudo-replicated, exploratory)	CD8^+^ T cell	8999	17460	0.0287	0.0193	HIGHER in High_SPP1_TAM			0.0525
sample level (inferential)	sample	23	24	0.0193	0.0973	LOWER in High_SPP1_TAM			0.315
sample-level SPP1^+^ fraction vs. mean CD8 exhaustion	sample					correlation (not a group comparison)	−0.0594	47	0.691

**Table 8:** Multi-level validation of the hypoxia–SPP1 association.

Level	Analysis	Statistical Unit	Method	r or Statistic	n	*p* Value
single cell	hypoxia score vs. SPP1 expression	macrophage	Spearman	0.1336	64,067	<2.2 × 10^−16^
single cell	hypoxia score, SPP1+ vs. SPP1- TAMs	macrophage	Wilcoxon		64,067	<2.2 × 10^−16^
single cell (sample level)	sample-level SPP1+ TAM fraction vs. mean macrophage hypoxia score	sample	Spearman	0.2343	47	0.113
spatial	hypoxia score vs. SPP1+ TAM signature	Visium section (8 sections)	Spearman per section; median	0.3424	8	0.00781
spatial	hypoxia score, hotspot vs. coldspot	Visium section (8 sections)	signed-rank over sections; median delta	0.0222	8	0.00781
TCGA-OV (bulk)	SPP1 expression vs. hypoxia score (TCGA-OV)	patient	Spearman	0.2034	429	2.28 × 10^−5^

## Data Availability

The single-cell RNA sequencing data analyzed in this study are publicly available from the GEO database under accession numbers GSE184880, GSE222556, and GSE241221; the Visium data used for spatial validation are available under accession number GSE263920. The TCGA-OV transcriptomic and clinical data used for external validation are publicly available through the Genomic Data Commons data portal. All intermediate result tables, figure panels, and code generated in this study are available from the corresponding author upon request.

## Supplementary Materials

The following supporting information can be downloaded at: https://www.mdpi.com/article/10.3390/ijms27219904/s1.

## Abbreviations

The following abbreviations are used in this manuscript:

TAMs	Tumor-associated macrophages
HGSOC	High-grade serous ovarian cancer
EMT	Epithelial–Mesenchymal Transition
TCGA-OV	The Cancer Genome Atlas—Ovarian Cancer
MAD	Median absolute deviation
PCA	Principal Component Analysis
UMAP	Uniform Manifold Approximation and Projection
GO	Gene Ontology
BP	Biological process
CC	Cellular component
MF	Molecular function
KEGG	Kyoto Encyclopedia of Genes and Genomes
GSEA	Gene set enrichment analysis
FDR	False Discovery Rate
